# Predicting mortality in febrile adults: comparative performance of the MEWS, qSOFA, and UVA scores using prospectively collected data among patients in four health-care sites in sub-Saharan Africa and South-Eastern Asia

**DOI:** 10.1016/j.eclinm.2024.102856

**Published:** 2024-10-04

**Authors:** Sham Lal, Manophab Luangraj, Suzanne H. Keddie, Elizabeth A. Ashley, Oliver Baerenbold, Quique Bassat, John Bradley, John A. Crump, Nicholas A. Feasey, Edward W. Green, Kevin C. Kain, Ioana D. Olaru, David G. Lalloo, Chrissy h. Roberts, David C.W. Mabey, Christopher C. Moore, Heidi Hopkins, the Febrile Illness Evaluation in a Broad Range of Endemicities (FIEBRE) Study Consortium, Sara Ajanovic, Benjamin Amos, Elizabeth A. Ashley, Oliver Baerenbold, Stéphanie Baghoumina, Núria Balanza, Tsitsi Bandason, Quique Bassat, Tapan Bhattacharyya, Stuart D. Blacksell, Zumilda Boca, Christian Bottomley, John Bradley, Justina M. Bramugy, Clare IR. Chandler, Vilada Chansamouth, Mabvuto Chimenya, Joseph Chipanga, Anelsio Cossa, John A. Crump, Ethel Dauya, Catherine Davis, Xavier de Lamballerie, Justin Dixon, Somyoth Douangphachanh, Audrey Dubot-Pérès, Michelle M. Durkin, Nicholas A. Feasey, Rashida A. Ferrand, Colin Fink, Elizabeth JA. Fitchett, Alessandro Gerada, Stephen R. Graves, Edward Green, Becca L. Handley, Heidi Hopkins, Coll D. Hutchison, Risara Jaksuwan, Jessica Jervis, Jayne Jones, Kevin C. Kain, Suzanne H. Keddie, Khamxeng Khounpaseuth, Katharina Kranzer, Khamfong Kunlaya, Pankaj Lal, Sham Lal, David G. Lalloo, Manophab Luangraj, Yoel Lubell, David CW. Mabey, Eleanor MacPherson, Forget Makoga, Sengchanh Manichan, Tegwen Marlais, Florian Maurer, Mayfong Mayxay, Michael Miles, Polycarp Mogeni, Campos Mucasse, Paul N. Newton, Chelsea Nguyen, Ioana D. Olaru, Vilayouth Phimolsarnnousith, Mathieu Picardeau, Chrissy H. Roberts, Amphone Sengduangphachanh, Siho Sengsavang, Molly Sibanda, Somvai Singha, John Stenos, Ampai Tanganuchitcharnchai, Hira Tanvir, James E. Ussher, Marta Valente, Marie A. Voice, Manivanh Vongsouvath, Msopole Wamaka, L Joseph Wheat, Shunmay Yeung

**Affiliations:** a Faculty of Infectious and Tropical Diseases, London School of Hygiene & Tropical Medicine, London, UK; b Lao-Oxford-Mahosot Hospital-Wellcome Trust Research Unit (LOMWRU), Mahosot Hospital, Vientiane, Lao PDR, Laos; c Faculty of Epidemiology and Population Health, London School of Hygiene & Tropical Medicine (LSHTM), London, UK; d Centre for Tropical Medicine and Global Health, University of Oxford, Oxford, UK; e ISGlobal, Hospital Clínic - Universitat de Barcelona, Barcelona, Spain; f Centre for International Health, University of Otago, Dunedin, New Zealand; g Department of Clinical Sciences, Liverpool School of Tropical Medicine, Liverpool, UK; h Malawi-Liverpool-Wellcome Trust Clinical Research Programme, Blantyre, Malawi; i Sandra Rotman Centre for Global Health, MaRS Centre, Department of Medicine, University Health Network-Toronto General Hospital, University of Toronto, 101 College St TMDT 10-360A, Toronto, ON M5G 1L7, Canada; j Department of Laboratory Medicine and Pathobiology, University of Toronto, Toronto, ON, Canada; k Division of Infectious Diseases and International Health, University of Virginia, Charlottesville, USA; l Centro de Investigação em Saúde de Manhiça (CISM), Maputo, Mozambique; m Pediatric Infectious Diseases Unit, Pediatrics Department, Hospital Sant Joan de Déu (University of Barcelona), Barcelona, Spain; n Division of Infectious Diseases, University Health Network, Toronto, ON, Canada; o ICREA, Pg. Lluís Companys 23, Barcelona 08010, Spain

**Keywords:** Severity scores, Prognostic scores, Mortality, qSOFA, MEWS, UVA, Fever, Area under the curve

## Abstract

**Background:**

Clinical severity scores can identify patients at risk of severe disease and death, and improve patient management. The modified early warning score (MEWS), the quick Sequential (Sepsis-Related) Organ Failure Assessment (qSOFA), and the Universal Vital Assessment (UVA) were developed as risk-stratification tools, but they have not been fully validated in low-resource settings where fever and infectious diseases are frequent reasons for health care seeking. We assessed the performance of MEWS, qSOFA, and UVA in predicting mortality among febrile patients in the Lao PDR, Malawi, Mozambique, and Zimbabwe.

**Methods:**

We prospectively enrolled in- and outpatients aged ≥ 15 years who presented with fever (≥37.5 °C) from June 2018–March 2021. We collected clinical data to calculate each severity score. The primary outcome was mortality 28 days after enrolment. The predictive performance of each score was determined using area under the receiver operating curve (AUC).

**Findings:**

A total of 2797 participants were included in this analysis. The median (IQR) age was 32 (24–43) years, 38% were inpatients, and 60% (1684/2797) were female. By the time of follow-up, 7% (185/2797) had died. The AUC (95% CI) for MEWS, qSOFA and UVA were 0.67 (0.63–0.71), 0.68 (0.64–0.72), and 0.82 (0.79–0.85), respectively. The AUC comparison found UVA outperformed both MEWS (p < 0.001) and qSOFA (p < 0.001).

**Interpretation:**

We showed that the UVA score performed best in predicting mortality among febrile participants by the time follow-up compared with MEWS and qSOFA, across all four study sites. The UVA score could be a valuable tool for early identification, triage, and initial treatment guidance of high-risk patients in resource-limited clinical settings.

**Funding:**

10.13039/501100020171FCDO.

**Research in context**

**Evidence before this study**

Before this study, there was limited evidence on the comparative performance of clinical severity scores to inform the prognosis of adult febrile patients presenting to hospitals and clinics in low-resource settings. Severity scores can be important for informing prompt management decisions, such as whether to admit the patient to hospital, the intensity of hospital management, diagnostic testing, and antibiotic therapy. However, most previous studies have derived severity scores and validated their performance in high-income settings and have been conducted at single centre sites among discrete patient populations. This approach may limit the application of severity scores to febrile populations in low-resource settings with different disease aetiologies and poorer access to diagnostics.

**Added value of this study**

We present evidence on the comparative performance of three adult clinical severity scores: the modified early warning score (MEWS), the quick Sequential (Sepsis-Related) Organ Failure Assessment (qSOFA), and the Universal Vital Assessment (UVA) in low-resource settings in four countries (Lao PDR, Malawi, Mozambique and Zimbabwe). Using data collected over the course of two years, this study demonstrates that the UVA score performed best across all four study sites in predicting mortality by the time of follow-up, compared with MEWS and qSOFA. We found UVA outperformed MEWS and qSOFA in both inpatients and outpatients. The inclusion of a patient’s HIV status improved the performance of MEWS and qSOFA, but UVA still outperformed MEWS and qSOFA.

**Implications of all the available evidence**

Based on these results, the UVA score could be used to support clinician’s prognosis for febrile patients in low-resource settings. It could help with early identification of patients who are at risk of severe disease and mortality and ensure they receive the appropriate level of care.

## Introduction

Fever is a common presenting symptom and reason for healthcare-seeking in low and middle-income countries (LMICs).^1^^,^^2^ While many febrile illnesses are self-resolving, some infectious causes of fever progress to sepsis and cause substantial morbidity and mortality if they are left untreated or are inadequately managed.^3^^,^^4^ Early identification of patients at high risk of clinical deterioration is critical to improving their management and averting poor outcomes. Clinical severity or prognostic scores can provide timely risk assessment of patients and guide urgent and increased level of care.^5^

The Modified Early Warning Score (MEWS) was adapted from the Early Warning Score (EWS) and designed for use in adolescents and adults in general wards upon patient admission. MEWS uses vital signs (Table 1) to identify critical illness. A score from 3 to 11 or higher is associated with a higher chance of mortality and intensive care unit (ICU) admission than a score below 3.^6^, ^7^, ^8^ The quick Sequential (sepsis-related) Organ Failure Assessment (qSOFA) was developed from the Sequential Organ Failure Assessment (SOFA) and consists of three criteria: blood pressure, respiratory rate, and Glasgow Coma Scale (GCS) score.^3^^,^^9^^,^^10^ The qSOFA has performed significantly better than the Systemic Inflammatory Response Syndrome (SIRS) criteria (heart rate, respiratory rate, temperature and abnormal white blood cell count) for predicting in-hospital death or admission to the ICU and is used for the evaluation of sepsis during hospital admission^11^, ^12^, ^13^, ^14^; however, it has limited sensitivity for predicting death.^15^^,^^16^

**Table 1:** Components of severity scores and their threshold values and points allocated for the Modified Early Warning Score (MEWS), quick Sequential Organ Failure Assessment (qSOFA) and Universal Vital Assessment scores (UVA).

Components of severity scores	MEWS range (0–11)		qSOFA (range 0–3)		UVA (range 0–12)	
	Threshold	Points	Threshold	Points	Threshold	Points
Body temperature (°C)	≥38.5	2			<36	2
	35–38.4	0			≥36	0
	<35	2				
Heart rate (beats per minute)	≥130	3			≥120	1
	111–129	2			<120	0
	101–110	1				
	51–100	0				
	41–50	1				
	<40	2				
Respiratory rate (breaths per minute)	≥30	3	≥22	1	≥30	1
	21–29	2	<22	0	<30	0
	15–20	1				
	9–14	0				
	<9	2				
Systolic blood pressure (mmHg)	<70	3	≤100	1	<90	1
	71–80 81–100 101–199	2 1 0	>100	0	≥90	0
Oxygen saturation (%)					<92	2
Level of consciousness (GCS, AVPU)	U	3	<15	1	<15	4
	P	2	= 15	0	=15	0
	V	1				
	A	0				
HIV status					Positive	2
					Negative/Unknown	0

GCS = Glasgow Coma Scale, AVPU= A = Alert, V = response on verbal stimuli P = response on pain stimuli, U = unresponsive.

MEWS and qSOFA were developed in high income settings and their performance in low-resource settings presents additional considerations due to limited availability of clinical and diagnostic tools, differences in the epidemiology of fever, and a relatively higher burden of HIV. Recently, the Universal Vital Assessment (UVA) score was developed from a cohort of inpatients hospitalised in six countries in sub-Saharan Africa.^17^ UVA relies on clinical signs that are easily available in resource-limited settings: body temperature, heart rate, respiratory rate, systolic blood pressure, level of consciousness, oxygen saturation, and HIV infection status. Single-centre evaluations in Africa and Asia have found that UVA outperformed MEWS and qSOFA in predicting in-hospital mortality and a meta-analysis of MEWS, qSOFA, SIRS, and UVA found that UVA performed best at predicting mortality.^17^, ^18^, ^19^, ^20^, ^21^, ^22^

Despite these findings, there is limited evidence on the most appropriate severity score for use in clinical sites in low-resource settings. Previous studies have either been retrospective in design or conducted at single sites, which may not be representative of other patient populations and have limited power to detect differences between scores. Previous studies were also unable to measure all the parameters for multiple severity scores in the same populations, thus limiting the ability to compare score performance.^19^ In this study we undertook a prospective multi-centre validation of MEWS, qSOFA, and UVA in predicting mortality by the time of follow-up among febrile adults who presented as inpatients or outpatients in four different clinical sites in low-resource settings as part of the Febrile Illness Evaluation of a Broad Range Endemicities (FIEBRE) study.^23^

## Methods

### Study design and participants

This analysis used data from the multicentre prospective observational FIEBRE study, which was conducted from 2018 to 2021 at four study sites, three in sub-Saharan Africa: rural southern Malawi, peri-urban southern Mozambique, and urban Zimbabwe; and one in South-eastern Asia: rural northern Lao PDR. Patients aged 15 years and older who sought care at each site’s recruiting health facilities were screened by trained clinical study staff. Patients were enrolled if they met the following criteria: tympanic or axillary temperature of ≥37.5 °C at presentation; not having been hospitalised or having undergone surgery in the previous month; and willingness and ability to provide demographic and clinical information, and clinical samples, at the time of enrolment and 28 days later. Additionally, for outpatients, selection criteria included being resident at the time of enrolment within the defined catchment area around the health facility. Outpatients with specific respiratory symptoms (cough AND at least one of the following: yellow or green sputum, blood in sputum) or with diarrhoeal symptoms (three or more loose stools per 24 h) were excluded from the FIEBRE study. This was due to pre-existing substantial evidence on the causes of fever from pneumonia and diarrhoea.^24^^,^^25^ Clinical management decisions, including whether to admit for inpatient care or to treat as an outpatient, were made by health facility staff according to routine practice. All participants gave written informed consent for participation in the study.

During enrolment, study staff collected demographic data, took a standardised clinical history, and performed a physical examination which included measuring the clinical signs for calculating MEWS, qSOFA, and UVA scores. The primary outcome for this analysis was a participant’s clinical outcome (alive or deceased) at a follow-up visit which was scheduled to occur on day 28 after enrolment (for practical and logistical reasons, the study protocol allowed assessment from 26 to 48 days after enrolment). Only participants with complete data for severity scores and follow-up data were included in the analyses. All data were collected using electronic case report forms and Open Data Kit (ODK).^26^ Details of the methods and the standard operating procedures are available from the protocol and the FIEBRE study website (https://doi.org/10.17037/PUBS.04652739).^23^

### Severity score calculation

Clinical signs were used to calculate MEWS, qSOFA, and UVA scores. Along with HIV status, as self-reported by the patient or from HIV point-of-care testing performed on the day of enrolment, the six clinical signs needed were: body temperature (°C), heart rate (beats per minute), respiratory rate (breaths per minute), systolic blood pressure (mmHg), oxygen saturation (%), and level of consciousness (GCS/Alert-Voice-Pain-Unconscious (AVPU) scores) (Table 1). A modified version of the GCS was used for this analysis, to accommodate the fact that the FIEBRE study collected simpler data on limb movements than is required for the conventional GCS.^27^ This modified GCS gave a movement score of 6 for patients who moved spontaneously and/or in response to verbal requests, 3 for patients who moved only in response to pain, and 1 for those not moving at all. The modified GCS was also converted to the AVPU scale for the MEWS calculation in this analysis (Table 1).

### Statistical analysis

Demographic characteristics and clinical signs were presented as medians with interquartile ranges (IQR) for non-normally distributed continuous variables, whilst proportions with 95% confidence intervals (CI) were used to describe discrete variables. Chi-squared tests were used to determine associations between categorical variables and non-missing follow-up vital status outcomes (alive or dead) and Mann–Whitney U tests were used to compare differences between continuous variables and outcomes. Univariate and multivariable logistic regression models were developed to identify the predictive effect of each severity score (MEWS, qSOFA, and UVA) after an *a priori* adjustment for covariates known to be associated with mortality. These covariates included participant age, sex, and HIV status (MEWS and qSOFA only; HIV status was not included in the adjusted and unadjusted UVA regression models, since HIV status is a component of the UVA score). Odds ratios (OR) and adjusted odd ratios (aOR) were reported alongside 95% CIs. To improve the model fit, severity score scales were grouped to aggregate outcomes in circumstances where there were fewer than five data points on any point on the scales. The lowest severity score was the reference category for the qSOFA and UVA models (score of 0), whilst for MEWS the reference was a score of 1. Complete case analyses were performed among those with non-missing data for severity scores and alive or dead outcomes at follow up. A predefined significance level of p < 0.05 was used.

The area under the receiver operating characteristic curves (AUC) was calculated along with the sensitivity, specificity, positive predictive value (PPV), and negative predictive value (NPV) with 95% CI using score cut-offs to determine each score’s predictive ability for mortality. The comparative performance of each score was assessed by the pairwise comparison of AUCs using methods recommended by DeLong et al.^28^ Each severity score’s performance was also examined in subgroups in each of the four sites and among inpatients and outpatients separately. Sensitivity analyses were also undertaken to explore the impact of missing data on the complete case analysis. In the first sensitivity analysis, those missing a follow-up outcome were assumed to be alive, and in a second analysis missing outcomes were set to dead. Lastly, due to the high HIV prevalence, we also undertook an exploratory analysis to add HIV status to MEWS and qSOFA scores. All analyses were performed using R Statistical Software (v4.1.2; R Core Team 2021) and the R code which fully reproduces the analyses is freely available from: https://github.com/SLGiHub/FIEBRE_Adult_severity_scores. Model development and validation was reported according to the TRIPOD guidelines (Supplementary Table S8).^29^

### Ethics approval

Ethics approval for the study was obtained from the London School of Hygiene & Tropical Medicine Research & Ethics committee and from each site-specific ethics committee: In Lao PDR, the National Ethics Committee for Health Research and the Oxford Tropical Research Ethics Committees; in Malawi the University of Malawi College of Medicine Research and Ethics Committee; in Mozambique the Comité Institucional de Bioética para a Saúde do Centro de Investigação em Saúde de Manhiça and the Comité Nacional de Bioética em Saúde de Moçambique; and in Zimbabwe the Medical Research Council of Zimbabwe.

### Role of the funding source

The FIEBRE study is funded by UK aid from the UK government; the views expressed, however, do not necessarily reflect the UK government’s official policies.

## Results

### Participant characteristics

**Fig. 1:**
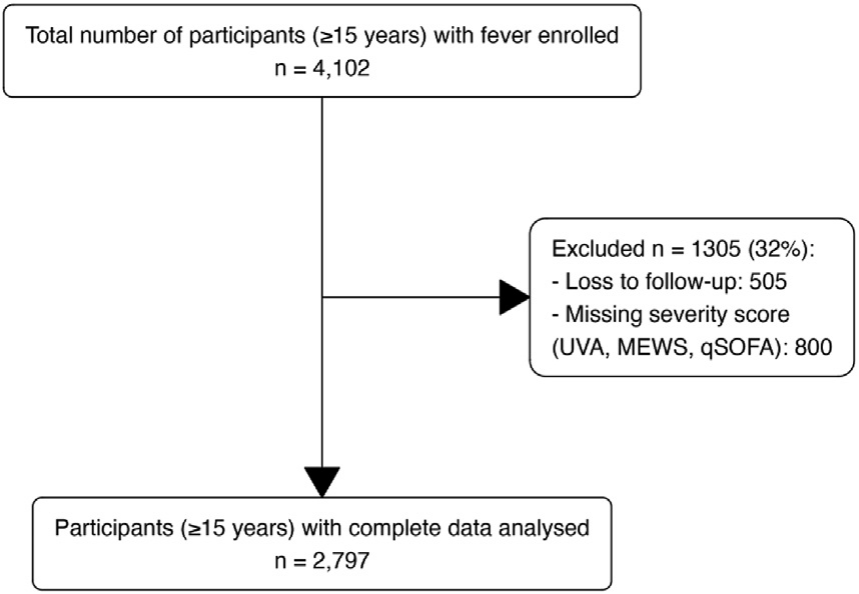
**Flowchart of febrile participants (aged** ≥ **15 years) enrolled and followed up between 2018 and 2021 at four sites (Lao PDR, Malawi, Mozambique, and Zimbabwe).** MEWS = modified early warning score, qSOFA = quick sequential organ failure assessment, UVA = universal vital assessment score.

This analysis included enrolled participants from all four FIEBRE sites, recruited over an approximately two-year period at each site from 25th June 2018 to 25th March 2021. A total of 4102 febrile participants were enrolled. Of 4102 participants, 505 (12%) were lost to follow-up, and 800 (20%) had missing data on HIV status or one or more component used to calculate severity scores (MEWS, qSOFA and UVA), leaving 2797 (68%) participants with complete data for this analysis (Fig. 1).

Across all sites, most participants were female (60% (1684/2797)), with a median (IQR) age of 32 (24–43) years. In Lao PDR 24% (100/412) of participants were aged 55 years and older, compared to 6% (47/727) in Malawi, 14% (114/811) in Mozambique and 9% (72/847) in Zimbabwe (Table 2). Mozambique had the highest HIV prevalence (51%, 413/811), whilst the HIV prevalence in Zimbabwe and Malawi was 20% (169/847) and 14% (102/727), respectively. In Lao PDR < 1% HIV prevalence was found through participant self-report; HIV diagnostic testing was not permitted by regulatory authorities at this site (Table 2). At all sites, a greater proportion of participants were enrolled as outpatients than inpatients (62% (1727/2797) vs. 38% (1070/2797), respectively) (Table 2). Among all inpatients, the median (IQR) length of hospital stay was 3 (2.0–6.0) days with limited variation across the sites (Table 2). The results of the three severity scores (MEWS, qSOFA, and UVA) are reported in Table 2. There were no substantial differences in characteristics between participants with complete and incomplete data (Supplementary Table S1).

**Table 2:** Demographic and clinical characteristics of febrile participants (aged ≥ 15 years) enrolled between 2018 and 2021 across four sites (Lao PDR, Malawi, Mozambique, and Zimbabwe), who had complete enrolment and follow-up data.

Variable	Lao PDR, N = 412	Malawi, N = 727	Mozambique, N = 811	Zimbabwe, N = 847	Overall, N = 2797
Age (years)					
Median (IQR)	36.0 (25.8, 54.0)	28.0 (22.0, 38.0)	34.0 (25.0, 45.5)	32.0 (23.0, 41.0)	32.0 (24.0, 43.0)
Age group, n (%)					
15–<25	92 (22%)	254 (35%)	184 (23%)	252 (30%)	784 (28%)
25–<35	100 (24%)	215 (30%)	217 (27%)	246 (29%)	782 (28%)
35–<40	64 (16%)	152 (21%)	188 (24%)	182 (21%)	590 (21%)
45–<55	56 (14%)	59 (8%)	90 (11%)	95 (11%)	301 (11%)
55–<65	49 (12%)	30 (4%)	73 (9%)	42 (5%)	197 (7%)
65+	51 (12%)	17 (2%)	41 (6%)	30 (4%)	143 (5%)
Sex, n (%)					
Female	234 (57%)	459 (63%)	521 (66%)	459 (54%)	1684 (60%)
Male	178 (43%)	268 (37%)	272 (34%)	388 (46%)	1113 (40%)
Patient group, n (%)					
Inpatient	178 (43%)	169 (23%)	352 (45%)	359 (42%)	1070 (38%)
Outpatient	234 (57%)	558 (77%)	441 (55%)	488 (58%)	1727 (62%)
Length of inpatient stay (days)^a^					
Median (IQR)	3.0 (1.0, 4.0)	3.0 (1.8, 4.0)	3.0 (1.0, 7.0)	5.0 (3.0, 8.5)	3.0 (2.0, 6.0)
Temperature (°C)					
Median (IQR)	38.0 (37.5, 38.5)	37.8 (37.6, 38.5)	38.0 (37.7, 38.6)	38.0 (37.8, 38.8)	38.0 (37.7, 38.6)
HIV status, n (%)					
Positive	1 (<1%)	102 (14%)	413 (51%)	169 (20%)	685 (24%)
Negative	411 (100%)	625 (86%)	398 (49%)	678 (80%)	2112 (76%)
Respiratory rate (brpm)					
Median (IQR)	22.0 (20.0, 25.0)	22.0 (20.0, 28.0)	20.0 (18.0, 20.0)	20.0 (18.0, 20.0)	20.0 (18.0, 23.0)
Systolic blood pressure (mmHg)					
Median (IQR)	120.0 (110.0, 125.0)	116.0 (106.0, 125.5)	117.0 (105.0, 130.0)	116.0 (105.0, 127.0)	117.0 (106.0, 127.0)
Heart rate (bpm)					
Median (IQR)	89.0 (80.0, 100.0)	95.0 (84.0, 109.0)	103.0 (90.0, 118.0)	106.0 (93.5, 120.0)	99.0 (87.0, 114.0)
Oxygen saturation (%)					
Median (IQR)	96.0 (95.0, 97.0)	97.0 (96.0, 98.0)	98.0 (96.0, 99.0)	95.0 (94.0, 97.0)	97.0 (95.0, 98.0)
MEWS					
Median (IQR)	3.0 (2.0, 4.0)	3.0 (2.0, 4.0)	3.0 (1.0, 4.0)	3.0 (2.0, 5.0)	3.0 (2.0, 4.0)
Modified GCS					
Median (IQR)	12.0 (12.0, 12.0)	12.0 (12.0, 12.0)	12.0 (12.0, 15.0)	12.0 (12.0, 12.0)	12.0 (12.0, 12.0)
qSOFA score, n (%)					
0	87 (21%)	275 (38%)	502 (62%)	572 (68%)	1436 (51%)
1	269 (65%)	372 (51%)	246 (31%)	239 (28%)	1126 (40%)
2	49 (12%)	80 (11%)	61 (8%)	36 (4%)	226 (8%)
3	7 (2%)	0 (0%)	2 (<1%)	0 (0%)	9 (<1%)
UVA score					
Median (IQR)	0.0 (0.0, 0.0)	0.0 (0.0, 1.0)	2.0 (0.0, 2.0)	0.0 (0.0, 2.0)	0.0 (0.0, 2.0)
Altered mental status (GCS < 15), n (%)					
Yes	15 (4%)	9 (1%)	45 (6%)	5 (1%)	74 (3%)
No	397 (96%)	718 (99%)	766 (94%)	842 (99%)	2723 (97%)

IQR = interquartile range, bpm = beats per minute, brpm = breaths per minute, MEWS = modified early warning score, GCS = Glasgow coma scale score, qSOFA = quick sequential organ failure assessment, UVA = universal vital assessment score.

a Length of hospital stay reported for inpatients only.

### Participant outcomes

Across all sites, 2458 (88%) of 2797 participants were discharged to home after enrolment and admission (Table 3). During the follow-up period, 185 deaths were reported, with an overall mortality of 7% (185/2779) (Table 3). Mortality varied across the sites, with the highest proportion of deaths reported in Mozambique (12% (95/811)), followed by Lao PDR (6% (23/412)), Zimbabwe (6%, (51/847)) and Malawi (2% (16/727)) (Table 3). There was also a marked association between increasing mortality and older age group (p < 0.001), which appeared to be driven by mortality in older age groups in Lao PDR and Mozambique (Supplementary Table S2). Mortality was similar across age groups in Malawi, whilst in Zimbabwe it was highest in the 45 to <55 age group (15%, 14/95) (Supplementary Table S2). The median (IQR) time to death after presentation was 10 (IQR 3–21) days and similar across all sites (Supplementary Fig. S1). There was a non-significant difference in the median time to death among participants who were enrolled as outpatients compared to inpatients, (2 vs. 10 days respectively, p = 0.069, Supplementary Table S3). Mortality was higher among males vs. females (9% vs. 5%, p < 0.001), inpatients vs. outpatients (16% vs. 1%, p < 0.001), and participants living with HIV vs. participants living without HIV (17% vs. 3%, p < 0.001).

**Table 3:** Demographic and clinical characteristics of febrile participants (aged ≥ 15 years) enrolled between 2018 and 2021 across four sites (Lao PDR, Malawi, Mozambique, and Zimbabwe), stratified by outcome by time of follow-up.

Variable	Alive at follow-up, N = 2612 (93%)	Dead at follow-up, N = 185 (7%)	Overall, N = 2797
Age group, n (%)			
15–<25	769 (98%)	15 (2%)	784
25–<35	740 (95%)	42 (5%)	782
35–<40	545 (92%)	45 (8%)	590
45–<55	269 (89%)	32 (11%)	301
55–<65	169 (86%)	28 (14%)	197
65+	120 (84%)	23 (16%)	143
Sex, n (%)			
Female	1600 (95%)	84 (5%)	1684
Male	1012 (91%)	101 (9%)	1113
Patient group, n (%)			
Inpatient	900 (84%)	170 (16%)	1070
Outpatient	1712 (99%)	15 (1%)	1727
Length of inpatient stay (days)^a^			
Median (IQR)	3.0 (2.0, 6.0)	3.5 (1.0, 7.0)	
Site, n (%)			
Lao PDR	389 (94%)	23 (6%)	412
Malawi	711 (98%)	16 (2%)	727
Mozambique	716 (88%)	95 (12%)	811
Zimbabwe	796 (94%)	51 (6%)	847
Temperature (°C)			
Median (IQR)	38.0 (37.7, 38.6)	38.1 (37.8, 38.8)	
HIV status, n (%)			
Positive	573 (84%)	112 (17%)	675
Negative	2039 (97%)	73 (3%)	2104
Respiratory rate (brpm)			
Median (IQR)	20.0 (18.0, 23.0)	22.0 (20.0, 26.0)	
Systolic blood pressure (mmHg)			
Median (IQR)	117.0 (106.0, 127.0)	111.0 (98.0, 129.0)	
Heart rate (bpm)			
Median (IQR)	99.0 (87.0, 113.0)	110.0 (94.0, 125.0)	
Oxygen saturation (%)			
Median (IQR)	97.0 (95.0, 98.0)	95.0 (92.0, 97.0)	
MEWS			
Median (IQR)	3.0 (2.0, 4.0)	4.0 (3.0, 6.0)	
Modified GCS			
Median (IQR)	15.0 (15.0, 15.0)	15.0 (15.0, 15.0)	
qSOFA score, n (%)			
0	1387 (97%)	49 (3%)	1436
1	1047 (93%)	79 (7%)	1126
2	176 (78%)	50 (22%)	226
3	2 (22%)	7 (78%)	9
UVA score			
Median (IQR)	0.0 (0.0, 2.0)	3.0 (2.0, 4.0)	
Altered mental status (Glasgow Coma Scale < 15), n (%)^b^			
Yes	38 (5%)	36 (20%)	74
No	2574 (95%)	149 (80%)	2723
Time to death (days)			
Median (IQR)	n/a	10.0 (3.0, 21.0)	
(Missing)	n/a	29	
In-hospital outcomes, n (%)			
Died	0 (0%)	73 (100%)	73
Discharge home	2458 (98%)	61 (2%)	2519
Discharged to palliative care	12 (54%)	11 (46%)	23
Other	19 (90%)	2 (10%)	21
Referred other	63 (67%)	30 (33%)	93
(Missing)	60	8	68

IQR = interquartile range, bpm = beats per minute, MEWS = modified early warning score, GCS = Glasgow coma scale score, qSOFA = quick sequential organ failure assessment, UVA = universal vital assessment score.

a Length of stay reported for inpatients only.

b Percentages for altered mental status are presented as column percentages, rather than row percentages.

**Fig. 2:**
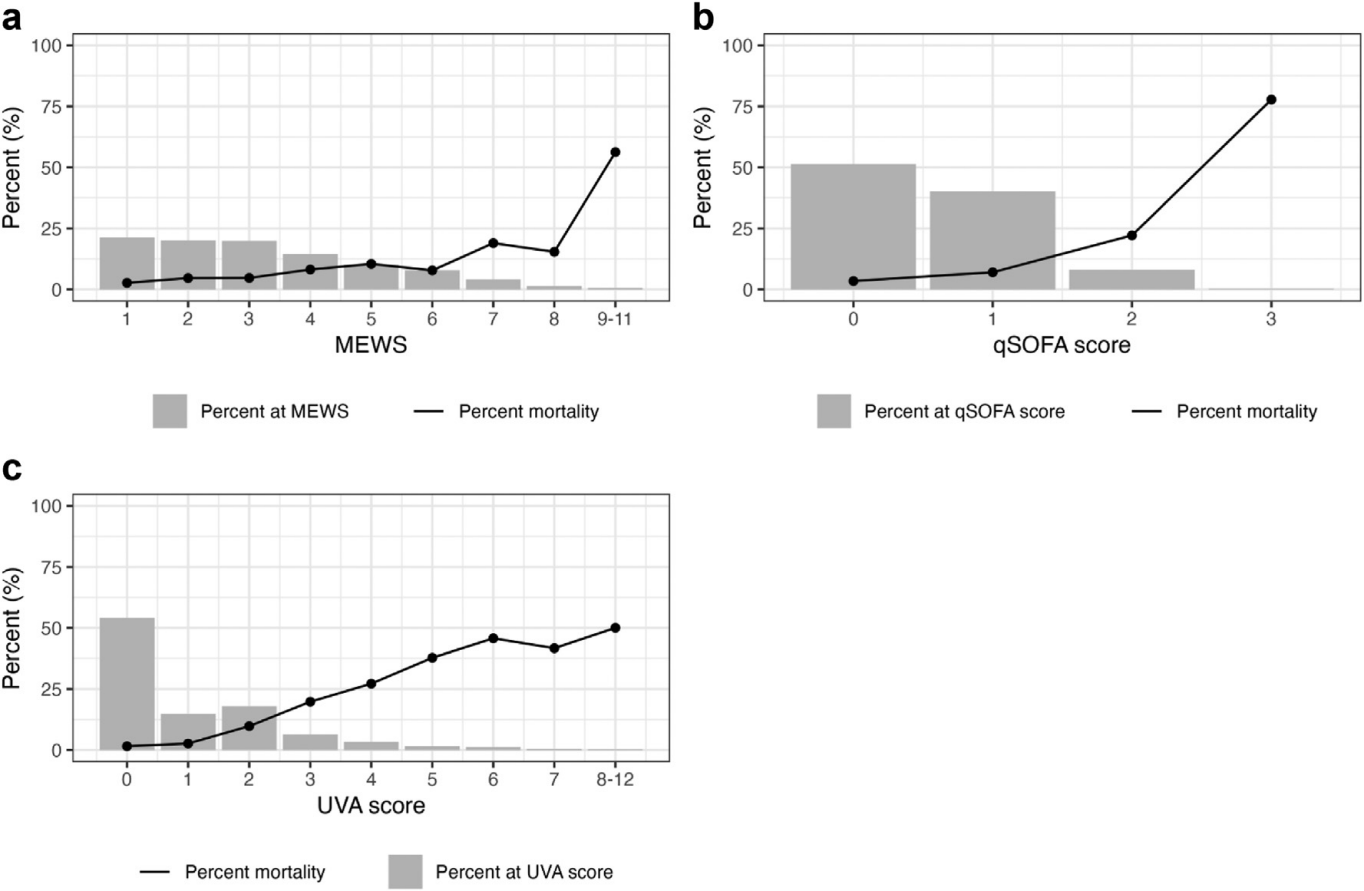
**The percentage distribution of each severity score (MEWS, qSOFA and UVA) and associated mortality at time of day 28 follow-up, among febrile participants (aged** ≥ **15 years) with complete data enrolled between 2018 and 2021 across four sites (Lao PDR, Malawi, Mozambique, and Zimbabwe).** Severity scores above 8 for MEWS and above 7 for UVA were grouped due to less than 5 outcomes reported. MEWS = modified early warning score, qSOFA = quick sequential organ failure assessment, UVA = universal vital assessment score.

Severity scores were higher among participants who died by the time of follow-up for each of the scores; mortality was 78% among those with a qSOFA score of 3, compared with 3% among those with a qSOFA score of 0. Similarly, the median MEWS and UVA scores were higher amongst those who died compared with those who survived (Table 3). Fig. 2a–c and show an increasing severity score and an increasing proportion of deaths by the time of follow-up. In addition, mortality for each severity score by site is shown in Supplementary Table S4.

### Predictors associated with mortality at follow-up

The unadjusted analyses for MEWS and qSOFA found age, sex, and living with HIV were all associated with a higher odds of death by the time of follow-up (Supplementary Tables S5 and S6). The unadjusted models for UVA also found age and sex associated with a higher odds of death (Supplementary Table S7). A similar pattern was observed in the adjusted analyses. Supplementary Fig. S2 shows a higher odds of death for an increase in severity score, after adjusting for age, sex, and HIV status (MEWS and qSOFA only). The adjusted analysis for each score also found increasing age and male sex were associated with mortality independently of the severity score (Supplementary Tables S5–S7). Living with HIV was also independently associated with mortality; participants who were living with HIV had a 5-fold greater odds of death compared with participants who were not, after controlling for severity score, age and sex (MEWS, adjusted OR (aOR) 4.92, 95% CI 3.49–6.98, and qSOFA, aOR 5.62, 95% CI 3.98–7.99) (Supplementary Tables S5–S7).

### Performance of severity scores (MEWS, qSOFA and UVA)

**Fig. 3:**
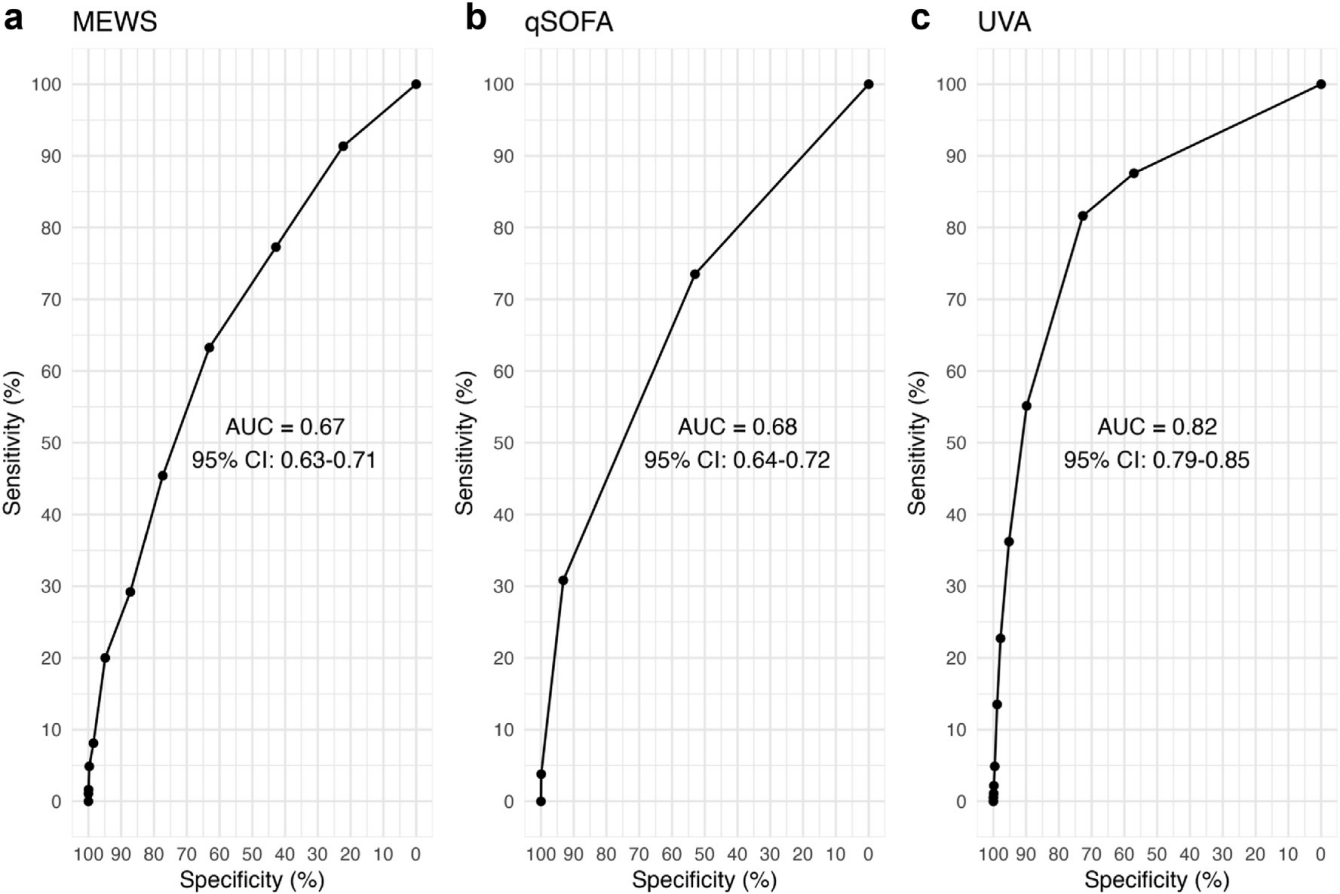
**ROC curves for a) MEWS, b) qSOFA and c) UVA severity scores for predicting mortality by time of follow-up among febrile participants (aged** ≥ **15 years) enrolled between 2018 and 2021 across four sites (Lao PDR, Malawi, Mozambique, and Zimbabwe).** ROC = receiver operating characteristic, AUC = area under the curve, MEWS = modified early warning score, qSOFA = quick sequential organ failure assessment, UVA = universal vital assessment score, CI = confidence interval.

The AUCs for each severity score were: MEWS, 0.67 (95%CI 0.63–0.71); qSOFA, 0.68 (95%CI 0.64–0.72); and UVA, 0.82 (95%CI 0.79–0.85) (Fig. 3). The pairwise comparison using the DeLong test found UVA outperformed both MEWS (AUC 0.82 vs. AUC 0.67, p < 0.001) and qSOFA (AUC 0.82 vs. AUC 0.68, p < 0.001). There was poor evidence for a difference between MEWS and qSOFA (AUC 0.67 vs. AUC 0.68, p = 0.527) in their performance (Table 5). The sensitivity, specificity, PPV, and NPV estimated for each score threshold are shown in Table 4.

**Table 5:** Comparative performance of MEWS, qSOFA and UVA scores and subgroup analyses in predicting mortality at time of follow-up of participants (aged ≥ 15 years) across four sites (Lao PDR, Malawi, Mozambique, and Zimbabwe).

	Severity score AUC (95% CI)			AUC pairwise comparison DeLong test		
	MEWS	qSOFA	UVA	UVA vs. MEWS	UVA vs. qSOFA	MEWS vs. qSOFA
Complete case analysis	0.67 (0.63–0.71)	0.68 (0.64–0.72)	0.82 (0.79–0.85)	p < 0.001	p < 0.001	p = 0.527
*Sensitivity analysis*						
Missing outcome set to alive	0.66 (0.62–0.70)	0.68 (0.64–0.72)	0.81 (0.78–0.85)	p < 0.001	p < 0.001	p = 0.311
Missing outcome set to dead	0.57 (0.55–0.60)	0.53 (0.51–0.56)	0.63 (0.60–0.65)	p < 0.001	p < 0.001	p = 0.001
*Subgroup analyses*						
Inpatients	0.62 (0.57–0.67)	0.64 (0.60–0.69)	0.75 (0.72–0.79)	p < 0.001	p < 0.001	p = 0.353
Outpatients	0.64 (0.53–0.76)	0.59 (0.46–0.72)	0.76 (0.61–0.91)	p = 0.054	p = 0.052	p = 0.573
Lao PDR	0.72 (0.61–0.82)	0.82 (0.74–0.90)	0.85 (0.75–0.94)	p = 0.020	p = 0.546	p = 0.013
Malawi	0.74 (0.59–0.88)	0.79 (0.68–0.90)	0.85 (0.73–0.96)	p = 0.116	p = 0.303	p = 0.386
Mozambique	0.70 (0.64–0.76)	0.74 (0.69–0.80)	0.76 (0.71–0.82)	p = 0.046	p = 0.483	p = 0.119
Zimbabwe	0.57 (0.50–0.65)	0.60 (0.53–0.68)	0.82 (0.77–0.87)	p < 0.001	p < 0.001	p = 0.446

AUC = Area under the curve, MEWS = modified early warning score, qSOFA = quick sequential organ failure assessment, UVA = universal vital assessment score, CI = confidence intervals.

**Table 4:** Sensitivity, specificity, negative predictive value (NPV) and positive predictive value (PPV) values estimated from receiver operating characteristic curves among febrile participants (aged ≥ 15 years) enrolled between 2018 and 2021 with complete data by time of follow-up across four countries (Lao PDR, Malawi, Mozambique, and Zimbabwe).

Threshold	Specificity	Sensitivity	NPV	PPV
MEWS				
0	0.00	100.00		6.61
1	22.32	91.35	97.33	7.69
2	42.84	77.30	96.38	8.74
3	63.09	63.24	96.04	10.82
4	77.34	45.41	95.24	12.43
5	87.17	29.19	94.56	13.88
6	94.87	20.00	94.36	21.64
7	98.47	8.11	93.80	27.27
8	99.73	4.86	93.67	56.25
9	99.96	1.62	93.48	75.00
10	100.00	1.08	93.45	100.00
11	100.00	0.00	93.39	
qSOFA score				
0	0.00	100.00		6.61
1	53.10	73.51	96.59	9.99
2	93.19	30.81	95.00	24.26
3	99.92	3.78	93.62	77.78
4	100.00	0.00	93.39	
UVA score				
0	0.00	100.00		6.61
1	56.97	87.57	98.48	12.60
2	72.47	81.62	98.24	17.36
3	89.78	55.14	96.58	27.64
4	95.21	36.22	95.47	34.90
5	97.78	22.70	94.70	42.00
6	98.85	13.51	94.16	45.45
7	99.58	4.86	93.66	45.00
8	99.85	2.16	93.51	50.00
9	99.92	1.08	93.45	50.00
10	100.00	0.54	93.42	100.00
11	100.00	0.00	93.39	

MEWS = modified early warning score, qSOFA = quick sequential organ failure assessment, UVA = universal vital assessment score, NPV = negative predictive value, PPV = positive predictive value.

A sensitivity analysis was undertaken to determine whether each score’s performance was affected by missing outcome data (Table 5). If all participants with missing outcome data were assumed to be alive by follow-up, UVA had a higher discriminant ability and higher AUC than MEWS (AUC 0.81 vs. AUC 0.66, p < 0.001) and qSOFA (AUC 0.81 vs. AUC 0.68, p < 0.001, Supplementary Fig. S3). There was poor evidence for a difference between the performance of MEWS and qSOFA (AUC 0.66 vs. AUC 0.68, p = 0.311) (Table 5). A second sensitivity analysis, where participants with missing outcomes were assumed to have died, found lower AUCs for all scores when compared to the sensitivity analysis which assumed all participants survived (Table 5). However, UVA outperformed MEWS (AUC 0.63 vs. AUC 0.57, p < 0.001) and qSOFA (AUC 0.63 vs. AUC 0.53, p < 0.001), while MEWS outperformed qSOFA (AUC 0.57 vs. AUC 0.53, p = 0.001) (Supplementary Fig. S4, Table 5).

Among inpatients, UVA also performed better than both MEWS (AUC 0.75 vs. AUC 0.62, p < 0.001) and qSOFA (AUC 0.75 vs. AUC 0.64, p < 0.001), but there was poor evidence of a difference between MEWS and qSOFA (AUC 0.62 vs. AUC 0.64, p = 0.353) (Supplementary Fig. S5, Table 5). Similarly for outpatients, Table 5 shows a higher predictive performance of UVA compared with MEWS (AUC 0.76 vs. AUC 0.64, p = 0.054) and a higher performance of UVA compared with qSOFA (AUC 0.76 vs. AUC 0.59, p = 0.052) (Supplementary Fig. S6). A subgroup analysis in each site found variable performance of the scores. In Lao PDR (Supplementary Fig. S7), Mozambique (Supplementary Fig. S9) and Zimbabwe (Supplementary Fig. S10) UVA performed better than MEWS (AUC 0.85 vs. AUC 0.72, p = 0.020 and AUC 0.76 vs. AUC 0.70, p = 0.046 and AUC 0.82 vs. AUC 0.57, p < 0.001 respectively). In Zimbabwe UVA outperformed qSOFA (AUC 0.82 vs. AUC 0.60, p < 0.001). There was poor evidence of a difference between UVA and qSOFA scores in Lao PDR, Malawi and Mozambique (Table 5).

An additional exploratory analysis was undertaken to assess the predictive abilities of MEWS and qSOFA with HIV status added to each score. Adding HIV status to MEWS increased its predictive performance when compared to MEWS without HIV status (AUC 0.74 vs. AUC 0.67, p < 0.001) (Supplementary Fig. S11). However, UVA still outperformed MEWS with HIV (AUC 0.82 vs. AUC 0.74, p < 0.001). Similarly, Supplementary Fig. S12 shows that adding HIV status to qSOFA increased its predictive performance compared to qSOFA without HIV status (AUC 0.78 vs. AUC 0.68, p < 0.001). Again, there was evidence that UVA still outperformed qSOFA with HIV (AUC 0.82 vs. AUC 0.78, p = 0.008). Lastly, qSOFA with HIV status outperformed MEWS with HIV status (AUC 0.78 vs. AUC 0.74, p = 0.023).

## Discussion

In this study we compared the performance of three severity scores, MEWS, qSOFA and UVA, to predict mortality amongst adult febrile patients in four sites in Africa and South-eastern Asia. The comparative analysis found that UVA outperformed both MEWS and qSOFA for identifying patients likely to die by end of follow-up. A sub-group analysis for inpatients and outpatients found this result was consistent for inpatients and outpatients. There was minimal difference in performance between MEWS and qSOFA. Whilst patient age, sex, and HIV infection status were all associated with mortality, for all three scores logistic regression models found that odds of death increased as severity scores increased, independently of HIV status, age and sex.

There was some heterogeneity in the performance of UVA compared to MEWS and qSOFA across study sites. In all sites except Malawi, UVA predicted mortality better than MEWS. In Zimbabwe, UVA predicted mortality better than qSOFA, whilst there was no significant difference between UVA and qSOFA in Lao PDR, Malawi, and Mozambique. A possible explanation could be that Zimbabwe had the highest proportion of deaths among participants living with HIV compared to all other sites, and UVA was the only score to include HIV status in its scoring system. Despite the lack of HIV testing for patients in Lao PDR, UVA performed better than MEWS at that site, consistent with results from a similar study in neighbouring Myanmar.^22^

The greater predictive ability of UVA over qSOFA could be due to different thresholds for each parameter and the inclusion of HIV status. The UVA score has a lower threshold for systolic blood pressure than qSOFA (90 vs. 100 mmHg) and a higher threshold for respiratory rate (30 vs. 22 per minute), which are likely to increase PPV for death. In addition, the UVA score was derived using data from sub-Saharan Africa rather than the European or North American cohorts used for MEWS and qSOFA. A further analysis of qSOFA and MEWS when HIV infection status was included in their scores increased their performance; but UVA still performed better than MEWS with HIV or qSOFA with HIV.

The performance of UVA in this study is consistent with studies in Africa and Asia. UVA had a greater prognostic ability compared to MEWS and qSOFA in Gabon (AUC of UVA 0.90 vs. MEWS 0.72 vs. qSOFA 0.77, respectively).^18^ In Tanzania, UVA outperformed MEWS and qSOFA (AUC 0.76 vs. 0.66 vs. 0.70, respectively).^20^ Similarly, in Myanmar UVA performed better than qSOFA (AUC 0.85 vs. 0.79, respectively).^22^

The predictive abilities of MEWS and qSOFA in our study were similar to those in the previously published literature.^16^^,^^30^ Higher scores for both MEWS and qSOFA were associated with higher odds of mortality, but both were limited in their ability to accurately identify patients likely to die by end of follow-up. Plausible explanations of poorer predictive ability could be due to MEWS ability to prognosticate only inpatients. Both MEWS and qSOFA were also created from patients in high income settings where records and clinical infrastructure enable close monitoring of clinical signs over time, which is not often available in low-resource clinical settings.

This study has several strengths. We prospectively enrolled patients across a diversity of clinical settings in multiple countries in sub-Saharan Africa and South-eastern Asia over the course of two years. We included inpatients and outpatients from rural, peri-urban and urban settings, and from multiple clinical departments at each participating health care facility rather than selected emergency department or intensive care units (ICU) as is often reported in studies of prognostic score development and validation. Thus, this study provides evidence on the validity of these scores for diverse adult patient populations in typical clinical settings in low-resource settings. The study also used a standardised protocol and methodology to enrol and prospectively measure clinical signs for severity scores across all sites.

There were some limitations with this study. The inclusion criterion for patients was an elevated temperature (≥37.5 °C) at presentation, i.e. those with hypothermic or normal body temperatures were not enrolled. The scoring systems for both MEWS and UVA include temperature criteria, which could not be fully applied to our patient population which may limit our study’s results to hypothermic or normothermic patient populations. However, the UVA score may have performed even better had hypothermic patients been included. A sepsis study from Uganda showed that low body temperatures were more predictive of death than higher temperatures, and UVA assigns a higher score for low temperatures.^31^ We collected outcome data at the time of follow-up approximately from 28 to 48 days post enrolment during which time participants may have deteriorated due to complications unrelated to their initial presentation. Further implementation research could evaluate the performance of these scores to improve clinical management at the time of presentation. Another limitation of our study is the large proportion (32%) of participants with missing data, which consisted of participants who were lost to follow-up and to a larger extent those who were missing data for one or more components of the three severity scores (MEWS, qSOFA and UVA) on the day of enrolment. This may have introduced selection bias into the study population; however, we examined the history, demographics and available clinical signs of those with and without missing data and found both populations were similar. In addition, we undertook a sensitivity analysis of the performance of UVA amongst those lost to follow-up and found it still outperformed MEWS and qSOFA. We also found low mortality ratios in our study, despite these low ratios our sensitivity analysis demonstrated consistent prognostic UVA performance in high and low mortality scenarios. In addition, the performance of UVA was consistent with studies for the literature with similar patient mix and mortality ratio, Gabon (UVA AUC 0.90), Rwanda (UVA AUC 0.77), and Tanzania (UVA AUC 0.85).^18^^,^^20^^,^^21^

In this multi-centre study comparing the predictive performance of three clinical severity scores amongst adult febrile patients seeking health care in four clinical settings in sub-Saharan Africa and South-eastern Asia, we found that the UVA score had the greatest ability to predict mortality by follow-up compared with MEWS and qSOFA. HIV infection status is an integral component of the UVA scoring system; the addition of HIV status to MEWS and qSOFA improved their performance in these settings, but UVA was still the best predictor of mortality. The UVA score uses clinical data that are often obtainable in resource-limited clinical settings, and could improve early identification, triage, and treatment of adult patients at high risk of mortality in such contexts.

## Contributors

Contributors SL, ML, HH, JAC, CCM and DCWM conceived the study design. ML, EA, QB, NAF, EG, IDO, and HH oversaw study activities at the clinical sites where data were collected. SL and OB conducted the statistical analyses and SK and JB accessed and verified the data and provided statistical support. JAC, CCM, DCWM and HH interpreted the results. SL and ML prepared the first draft of the manuscript, which was reviewed and edited by SK, EA, QB, JB, JAC, NAF, CHR, KCK, IDO, DCWM, CCM, HH. All authors reviewed and approved the final manuscript.

## Data sharing statement

The de-identified dataset, along with the corresponding data dictionary that defines each field in the set, are freely available with no restrictions via LSHTM’s Data Compass and can be accessed and downloaded at https://github.com/SLGiHub/FIEBRE_Adult_severity_scores.

## Declaration of interests

HH reports royalties from Wolters Kluwer Health as the primary author and maintainer of the “Laboratory tools for diagnosis of malaria” clinical decision support tool.

## Appendix ASupplementary data

Supplementary Figs. S1–S11 and Tables S1–S8

FIEBRE-consortium-list-for-publications

## References

[1] Reyburn H., Mbatia R., Drakeley C. (2004). Overdiagnosis of malaria in patients with severe febrile illness in Tanzania: a prospective study. BMJ.

[2] Mayxay M., Castonguay-Vanier J., Chansamouth V. (2013). Causes of non-malarial fever in Laos: a prospective study. Lancet Glob Health.

[3] Singer M., Deutschman C.S., Seymour C.W. (2016). The third international consensus definitions for sepsis and septic shock (Sepsis-3). JAMA.

[4] Cecconi M., Evans L., Levy M., Rhodes A. (2018). Sepsis and septic shock. Lancet.

[5] Gerry S., Bonnici T., Birks J. (2020). Early warning scores for detecting deterioration in adult hospital patients: systematic review and critical appraisal of methodology. BMJ.

[6] Morgan R., Williams F., Wright M. (1997). An early warning scoring system for detecting developing critical illness. Clin Intensive Care.

[7] Quarterman C.P.J., Thomas A.N., McKenna M., McNamee R. (2005). Use of a patient information system to audit the introduction of modified early warning scoring. J Eval Clin Pract.

[8] Kruisselbrink R., Kwizera A., Crowther M. (2016). Modified early warning score (MEWS) identifies critical illness among ward patients in a resource restricted setting in kampala, Uganda: a prospective observational study. PLoS One.

[9] Solligård E., Damås J.K. (2017). SOFA criteria predict infection-related in-hospital mortality in ICU patients better than SIRS criteria and the qSOFA score. BMJ Evid Based Med.

[10] Spoto S., Nobile E., Carnà E.P.R. (2020). Best diagnostic accuracy of sepsis combining SIRS criteria or qSOFA score with Procalcitonin and Mid-Regional pro-Adrenomedullin outside ICU. Sci Rep.

[11] Bone R.C., Balk R.A., Cerra F.B. (1992). Definitions for sepsis and organ failure and guidelines for the use of innovative therapies in sepsis. Chest.

[12] Rudd K.E., Seymour C.W., Aluisio A.R. (2018). Association of the quick sequential (Sepsis-Related) organ failure assessment (qSOFA) score with excess hospital mortality in adults with suspected infection in low- and middle-income countries. JAMA.

[13] Mignot-Evers L., Raaijmakers V., Buunk G. (2021). Comparison of SIRS criteria and qSOFA score for identifying culture-positive sepsis in the emergency department: a prospective cross-sectional multicentre study. BMJ Open.

[14] Usul E., Korkut S., Kayipmaz A.E., Halici A., Kavalci C. (2021). The role of the quick sequential organ failure assessment score (qSOFA) and modified early warning score (MEWS) in the pre-hospitalization prediction of sepsis prognosis. Am J Emerg Med.

[15] Brabrand M., Havshøj U., Graham C.A. (2016). Validation of the qSOFA score for identification of septic patients: a retrospective study. Eur J Intern Med.

[16] Huson M.A.M., Katete C., Chunda L. (2017). Application of the qSOFA score to predict mortality in patients with suspected infection in a resource-limited setting in Malawi. Infection.

[17] Moore C.C., Hazard R., Saulters K.J. (2017). Derivation and validation of a universal vital assessment (UVA) score: a tool for predicting mortality in adult hospitalised patients in sub-Saharan Africa. BMJ Glob Health.

[18] Schmedding M., Adegbite B.R., Gould S. (2018). A prospective comparison of quick sequential organ failure assessment, systemic inflammatory response Syndrome criteria, universal vital assessment, and modified early warning score to predict mortality in patients with suspected infection in Gabon. Am J Trop Med Hyg.

[19] Adegbite B.R., Edoa J.R., Ndoumba W.F.N. (2021). A comparison of different scores for diagnosis and mortality prediction of adults with sepsis in Low-and-Middle-Income Countries: a systematic review and meta-analysis. EClinicalMedicine.

[20] Bonnewell J.P., Rubach M.P., Madut D.B. (2021). Performance assessment of the universal vital assessment score vs other illness severity scores for predicting risk of in-hospital death among adult febrile inpatients in northern Tanzania, 2016-2019. JAMA Netw Open.

[21] Klinger A., Mueller A., Sutherland T. (2021). Predicting mortality in adults with suspected infection in a Rwandan hospital: an evaluation of the adapted MEWS, qSOFA and UVA scores. BMJ Open.

[22] Mar Minn M., Aung N.M., Kyaw D.Z. (2021). The comparative ability of commonly used disease severity scores to predict death or a requirement for ICU care in patients hospitalised with possible sepsis in Yangon, Myanmar. Int J Infect Dis.

[23] Hopkins H., Bassat Q., Chandler C.I. (2020). Febrile illness evaluation in a Broad range of Endemicities (FIEBRE): protocol for a multisite prospective observational study of the causes of fever in Africa and Asia. BMJ Open.

[24] Kotloff K.L., Nataro J.P., Blackwelder W.C. (2013). Burden and aetiology of diarrhoeal disease in infants and young children in developing countries (the Global Enteric Multicenter Study, GEMS): a prospective, case-control study. Lancet.

[25] O’Brien K.L., Baggett H.C., Brooks W.A. (2019). Causes of severe pneumonia requiring hospital admission in children without HIV infection from Africa and Asia: the PERCH multi-country case-control study. Lancet.

[26] Marks M., Lal S., Brindle H. (2021). Electronic data management for vaccine trials in low resource settings: upgrades, scalability, and impact of ODK. Front Public Health.

[27] Teasdale G., Jennett B. (1974). Assessment of coma and impaired consciousness. A practical scale. Lancet.

[28] DeLong E.R., DeLong D.M., Clarke-Pearson D.L. (1988). Comparing the areas under two or more correlated receiver operating characteristic curves: a nonparametric approach. Biometrics.

[29] Moons K.G.M., Altman D.G., Reitsma J.B. (2015). Transparent reporting of a multivariable prediction model for individual prognosis or diagnosis (TRIPOD): explanation and elaboration. Ann Intern Med.

[30] Carugati M., Zhang H.L., Kilonzo K.G. (2018). Predicting mortality for adolescent and adult patients with fever in resource-limited settings. Am J Trop Med Hyg.

[31] Rice B., Calo S., Kamugisha J.B., Kamara N., Chamberlain S., Group on behalf of GECIS (2022). Emergency care of sepsis in sub-Saharan Africa: mortality and non-physician clinician management of sepsis in rural Uganda from 2010 to 2019. PLoS One.

