# Supplementary material for: Predicting mortality in febrile adults: comparative performance of the MEWS, qSOFA, and UVA scores using prospectively collected data among patients in four health-care sites in sub-Saharan Africa and South-Eastern Asia
Source: eClinicalMedicine. 2024 Oct 4;77:102856. doi: 10.1016/j.eclinm.2024.102856 (PMC11474423; doi:10.1016/j.eclinm.2024.102856)
Supplement: Supplementary Figs. S1–S11 and Tables S1–S8 [file mmc1.docx]

| Variable | Lao PDR, N = 800 | Malawi, N = 94 | Mozambique, N = 210 | Zimbabwe, N = 201 | Overall, N = 1,323^1^ |
| --- | --- | --- | --- | --- | --- |
| Age (years) |  |  |  |  |  |
| Median (IQR) | 35.0 (24.0, 54.0) | 28.0 (22.0, 39.0) | 33.0 (24.0, 47.0) | 28.0 (22.0, 38.0) | 33.0 (23.0, 50.0) |
| Age group, n (%) |  |  |  |  |  |
| 15 - <25 | 208 (26%) | 35 (37%) | 54 (26%) | 75 (37%) | 372 (29%) |
| 25 - <35 | 176 (22%) | 23 (24%) | 57 (27%) | 53 (26%) | 309 (24%) |
| 35 - <40 | 112 (14%) | 19 (20%) | 41 (20%) | 49 (24%) | 221 (17%) |
| 45 - <55 | 106 (13%) | 9 (9.6%) | 22 (10%) | 13 (7%) | 150 (11%) |
| 55 - <65 | 94 (12%) | 4 (4%) | 22 (10%) | 8 (4%) | 128 (10%) |
| 65+ | 104 (13%) | 4 (4%) | 14 (7%) | 3 (2%) | 125 (10%) |
| Sex, n (%) |  |  |  |  |  |
| Female | 41 (55%) | 42 (55%) | 128 (61%) | 94 (53%) | 692 (53%) |
| Male | 34 (45%) | 35 (45%) | 82 (39%) | 83 (47%) | 613 (47%) |
| Patient group, n (%) |  |  |  |  |  |
| Inpatient | 431 (54%) | 51 (54%) | 108 (51%) | 87 (43%) | 677 (52%) |
| Outpatient | 369 (46%) | 43 (46%) | 102 (49%) | 114 (57%) | 628 (48%) |
| Length of inpatient stay (days)* |  |  |  |  |  |
| Median (IQR) | 3.0 (2.0, 4.0) | 4.0 (1.0, 6.0) | 3.0 (1.0, 6.0) | 5.0 (3.0, 8.0) | 3.0 (2.0, 5.0) |
| (Missing) | 390 | 53 | 125 | 130 | 698 |
| Temperature (°C) |  |  |  |  |  |
| Median (IQR) | 37.9 (37.5, 38.5) | 38.2 (37.7, 38.9) | 38.1 (37.7, 38.7) | 38.1 (37.9, 38.8) | 38.0 (37.5, 38.5) |
| Range | 37.5, 40.9 | 37.5, 40.1 | 37.5, 40.0 | 37.5, 40.4 | 37.5, 40.9 |
| HIV status, n (%) |  |  |  |  |  |
| Positive | 0 (0%) | 16 (19%) | 83 (42%) | 43 (25%) | 142 (29%) |
| Negative | 31 (100%) | 68 (81%) | 106 (54%) | 132 (75%) | 337 (69%) |
| Indeterminant | 0 (0%) | 0 (0%) | 9 (5%) | 0 (0%) | 9 (2%) |
| Unknown | 769 | 10 | 12 | 26 | 817 |
| Respiratory rate (brpm) |  |  |  |  |  |
| Median (IQR) | 22.0 (20.0, 22.0) | 22.0 (19.0, 25.0) | 20.0 (18.0, 20.0) | 20.0 (18.0, 21.0) | 20.0 (20.0, 22.0) |
| (Missing) | 4 | 3 | 1 | 1 | 9 |
| Systolic blood pressure (mmHg) |  |  |  |  |  |
| Median (IQR) | 112.0 (100.0, 124.0) | 112.0 (103.0, 124.0) | 120.0 (106.0, 130.0) | 115.0 (102.0, 123.0) | 114.0 (101.3, 126.0) |
| (Missing) | 2 | 3 | 6 | 0 | 11 |
| Heart rate (bpm) |  |  |  |  |  |
| Median (IQR) | 90.0 (83.2, 100.0) | 101.0 (90.0, 113.5) | 99.0 (88.0, 116.0) | 106.0 (94.0, 121.0) | 96.0 (85.0, 108.0) |
| (Missing) | 2 | 0 | 0 | 0 | 2 |
| Oxygen saturation (%) |  |  |  |  |  |
| Median (IQR) | 96.0 (95.0, 98.0) | 96.0 (95.0, 98.0) | 98.0 (96.0, 99.0) | 98.0 (96.0, 99.0) | 96.0 (95.0, 98.0) |
| (Missing) | 15 | 2 | 5 | 7 | 1 |
| MEWS |  |  |  |  |  |
| Median (IQR) | 3.0 (2.0, 4.0) | 4.0 (3.0, 5.0) | 3.0 (1.0, 5.0) | 3.0 (2.0, 5.0) | 3.0 (2.0, 4.0) |
| (Missing) | 4 | 12 | 14 | 2 | 32 |
| Modified GCS |  |  |  |  |  |
| Median (IQR) | 15.0 (15.0, 15.0) | 15.0 (15.0, 15.0) | 15.0 (15.0, 15.0) | 15.0 (15.0, 15.0) | 15.0 (15.0, 15.0) |
| (Missing) | 1 | 1 | 5 | 1 | 7 |
| qSOFA score, n (%) |  |  |  |  |  |
| 0 | 547 (43%) | 32 (36%) | 128 (63%) | 132 (66%) | 560 (43%) |
| 1 | 559 (43%) | 40 (45%) | 58 (29%) | 53 (27%) | 563 (43%) |
| 2 | 170 (13%) | 16 (18%) | 17 (8.4%) | 14 (7%) | 171 (13%) |
| 3 | 10 (1%) | 0 (0%) | 0 (0%) | 0 (0%) | 10 (1%) |
| (Missing) | 19 | 6 | 7 | 2 | 19 |
| UVA score |  |  |  |  |  |
| Median (IQR) | 1.0 (0.0, 2.0) | 0.0 (0.0, 2.0) | 1.0 (0.0, 2.0) | 1.0 (0.0, 2.0) | 1.0 (0.0, 2.0) |
| (Missing) | 854 | 21 | 34 | 28 | 854 |
| Altered mental status   (GCS <15), n (%) |  |  |  |  |  |
| Yes | 24 (3%) | 7 (8%) | 9 (4%) | 1 (<1%) | 41 (3%) |
| No | 775 (97%) | 86 (92%) | 196 (96%) | 200 (100%) | 1,257 (97%) |
| (Missing) | 1 | 1 | 5 | 0 | 7 |
| In-hospital outcomes, n (%) |  |  |  |  |  |
| Died | 0 (0%) | 3 (4%) | 2 (1%) | 1 (0.5%) | 14 (1%) |
| Discharge home | 73 (97%) | 80 (93%) | 168 (85%) | 190 (99%) | 1,140 (90%) |
| Discharged to palliative care | 0 (0%) | 1 (1%) | 1 (<1%) | 0 (0%) | 40 (3%) |
| Other | 0 (0%) | 2 (2%) | 7 (4%) | 0 (0%) | 14 (1%) |
| Referred other | 2 (3%) | 0 (0%) | 19 (10%) | 1 (1%) | 65 (5%) |
| (Missing) | 0 | 8 | 13 | 9 | 32 |
| Day 28 outcome, n (%) |  |  |  |  |  |
| Alive | 672 (84%) | 17 (18%) | 26 (12%) | 23 (11%) | 738 (57%) |
| Dead | 53 (7%) | 2 (2%) | 6 (3%) | 1 (1%) | 62 (5%) |
| Lost to follow-up | 75 (99%) | 75 (80%) | 178 (85%) | 177 (88%) | 505 (39%) |

**Table S1 Demographic and clinical characteristics of febrile participants (aged ≥15 years) enrolled between 2018 and 2021 across four sites (Lao PDR, Malawi, Mozambique, and Zimbabwe)**, **who had incomplete enrolment and follow-up data.** IQR=interquartile range, bpm=beats per minute, brpm=breaths per minute, MEWS=modified early warning score, GCS=Glasgow coma scale score, qSOFA=quick sequential organ failure assessment, UVA=universal vital assessment score *Length of stay reported for inpatients only.

|  | Lao PDR | | | | Malawi | | | | Mozambique | | | | Zimbabwe | | | |
| --- | --- | --- | --- | --- | --- | --- | --- | --- | --- | --- | --- | --- | --- | --- | --- | --- |
| Variable | Alive at follow-up,  N = 389 | Dead at follow-up,  N = 23 | Overall,  N = 412 | Alive at follow-up,  N = 711 | | Dead at follow-up,  N = 16 | Overall,  N = 727 | Alive at follow-up,  N = 716 | | Dead at follow-up,  N = 95 | Overall,  N = 811 | Alive at follow-up,  N = 796 | | Dead at follow-up,  N = 51 | Overall,  N = 847 |  |
| Age group, n (%) |  |  |  |  | |  |  |  | |  |  |  | |  |  |  |
| 15 - <25 | 91 (99%) | 1 (1%) | 92 | 253 (100%) | | 1 (<1%) | 254 | 179 (96%) | | 7 (3.8%) | 186 | 246 (98%) | | 6 (2%) | 252 |  |
| 25 - <35 | 99 (99%) | 1 (1%) | 100 | 211 (98%) | | 4 (2%) | 215 | 199 (90%) | | 22 (10%) | 221 | 231 (94%) | | 15 (6%) | 246 |  |
| 35 - <40 | 62 (97%) | 2 (3%) | 64 | 147 (97%) | | 5 (3%) | 152 | 163 (85%) | | 29 (15%) | 192 | 173 (95%) | | 9 (5%) | 182 |  |
| 45 - <55 | 51 (91%) | 5 (9%) | 56 | 56 (95%) | | 3 (5%) | 59 | 81 (89%) | | 10 (11%) | 91 | 81 (85%) | | 14 (15%) | 95 |  |
| 55 - <65 | 43 (88%) | 6 (12%) | 49 | 29 (97%) | | 1 (3%) | 30 | 58 (76%) | | 18 (24%) | 76 | 39 (93%) | | 3 (7%) | 42 |  |
| 65+ | 43 (84%) | 8 (16%) | 51 | 15 (88%) | | 2 (12%) | 17 | 36 (80%) | | 9 (20%) | 45 | 26 (87%) | | 4 (13%) | 30 |  |
| Sex, n (%) |  |  |  |  | |  |  |  | |  |  |  | |  |  |  |
| Female | 223 (95%) | 11 (5%) | 234 | 454 (99%) | | 5 (1%) | 459 | 486 (91%) | | 46 (9%) | 532 | 437 (95%) | | 22 (5%) | 459 |  |
| Male | 166 (93%) | 12 (7%) | 178 | 257 (96%) | | 11 (4%) | 268 | 230 (82%) | | 49 (18%) | 279 | 359 (93%) | | 29 (8%) | 388 |  |
| Patient group, n (%) |  |  |  |  | |  |  |  | |  |  |  | |  |  |  |
| Inpatient | 159 (89%) | 19 (11%) | 178 | 153 (91%) | | 16 (10%) | 169 | 276 (76%) | | 88 (24%) | 364 | 312 (87%) | | 47 (13%) | 359 |  |
| Outpatient | 230 (98%) | 4 (7%) | 234 | 558 (100%) | | 0 (0%) | 558 | 440 (98%) | | 7 (2%) | 447 | 484 (99%) | | 4 (<1%) | 488 |  |
| HIV status, n (%) |  |  |  |  | |  |  |  | |  |  |  | |  |  |  |
| Positive | 1 (100%) | 0 (0%) | 1 | 94 (92%) | | 8 (8%) | 102 | 349 (85%) | | 64 (15%) | 413 | 129 (76%) | | 40 (24%) | 169 |  |
| Negative | 388 (94%) | 23 (6%) | 411 | 617 (99%) | | 8 (1%) | 625 | 367 (92%) | | 31 (8%) | 398 | 667 (98%) | | 11 (2%) | 678 |  |

**Table** **S2: Demographic characteristics of febrile participants (aged ≥15 years) enrolled between 2018 and 2021, stratified by outcome by site and time of follow-up.**

|  | Inpatient, N = 170 | Outpatient, N = 15 | Overall, N = 185 | p-value^1^ |
| --- | --- | --- | --- | --- |
| Time to death (days) |  |  |  | 0.069 |
| Median (IQR) | 10.0 (3.0, 22.0) | 2.0 (1.0, 20.0) | 10.0 (3.0, 21.0) |  |
| Range | 0.0, 82.0 | 0.0, 51.0 | 0.0, 82.0 |  |
| (Missing) | 27 | 2 | 29 |  |
|  | | | | |

**Table S3: Time to death (days) by time to follow-up by patient group among febrile participants (aged ≥15 years) enrolled between 2018 and 2021 across four sites (Lao PDR, Malawi, Mozambique, and Zimbabwe).** IQR=interquartile range, 1 = Mann-Whitney U test.

|  | Lao PDR | | | Malawi | | | | Mozambique | | | | Zimbabwe | | |
| --- | --- | --- | --- | --- | --- | --- | --- | --- | --- | --- | --- | --- | --- | --- |
| Severity score | Alive at follow-up, N = 389 | Dead at follow-up, N = 23 | Overall,  N = 412 | Alive at follow-up, N = 711 | Dead at follow-up,  N = 16 | Overall,  N = 727 | Alive at follow-up,  N = 716 | | Dead at follow-up,  N = 95 | Overall,  N = 811 | Alive at follow-up,  N = 796 | | Dead at follow-up,  N = 51 | Overall,  N = 847 |
| MEWS, n (%) |  |  |  |  |  |  |  | |  |  |  | |  |  |
| 1 | 58 (100%) | 0 (0%) | 58 | 139 (100%) | 0 (0%) | 139 | 215 (96%) | | 9 (4%) | 224 | 171 (96%) | | 7 (4%) | 178 |
| 2 | 129 (97%) | 4 (3%) | 133 | 171 (98%) | 4 (2%) | 175 | 115 (90%) | | 13 (10%) | 128 | 121 (96%) | | 5 (4%) | 126 |
| 3 | 53 (91%) | 5 (9%) | 58 | 151 (99%) | 2 (1%) | 153 | 151 (94%) | | 9 (6%) | 160 | 174 (95%) | | 10 (5%) | 184 |
| 4 | 99 (96%) | 4 (4%) | 103 | 81 (99%) | 1 (1%) | 82 | 82 (85%) | | 15 (15%) | 97 | 110 (89%) | | 13 (11%) | 123 |
| 5 | 27 (87%) | 4 (13%) | 31 | 71 (100%) | 0 (0%) | 71 | 55 (75%) | | 18 (25%) | 73 | 104 (93%) | | 8 (7%) | 112 |
| 6 | 16 (89%) | 2 (11%) | 18 | 57 (98%) | 1 (2%) | 58 | 55 (83%) | | 11 (17%) | 66 | 73 (96%) | | 3 (4%) | 76 |
| 7 | 5 (83%) | 1 (17%) | 6 | 28 (90%) | 3 (10%) | 31 | 27 (68%) | | 13 (33%) | 40 | 34 (87%) | | 5 (13%) | 39 |
| 8 | 2 (100%) | 0 (0%) | 2 | 11 (92%) | 1 (8%) | 12 | 12 (71%) | | 5 (29%) | 17 | 8 (100%) | | 0 (0%) | 8 |
| 9 | 0 (0%) | 3 (100%) | 3 | 1 (33%) | 2 (67%) | 3 | 4 (80%) | | 1 (20%) | 5 | 1 (100%) | | 0 (0%) | 1 |
| 10 | 0 | 0 | 0 | 1 (100%) | 0 (0%) | 1 | 0 (0%) | | 1 (100%) | 1 | 0 | | 0 | 0 |
| 11 | 0 | 0 | 0 | 0 (0%) | 2 (100%) | 2 | 0 | | 0 | 0 | 0 | | 0 | 0 |
| qSOFA score, n (%) |  |  |  |  |  |  |  | |  |  |  | |  |  |
| 0 | 87 (100%) | 0 (0%) | 87 | 274 (100%) | 1 (<1%) | 275 | 479 (95%) | | 23 (5%) | 502 | 547 (96%) | | 25 (4%) | 572 |
| 1 | 261 (97%) | 8 (3%) | 269 | 366 (98%) | 6 (2%) | 372 | 202 (82%) | | 44 (18%) | 246 | 218 (91%) | | 21 (9%) | 239 |
| 2 | 39 (80%) | 10 (20%) | 49 | 71 (89%) | 9 (11%) | 80 | 35 (57%) | | 26 (43%) | 61 | 31 (86%) | | 5 (14%) | 36 |
| 3 | 2 (29%) | 5 (71%) | 7 | 0 | 0 | 0 | 0 (0%) | | 2 (100%) | 2 | 0 | | 0 | 0 |
| UVA score, n (%) |  |  |  |  |  |  |  | |  |  |  | |  |  |
| 0 | 355 (98%) | 6 (2%) | 361 | 406 (100%) | 2 (<1%) | 408 | 282 (96%) | | 11 (4%) | 293 | 445 (99%) | | 4 (1%) | 449 |
| 1 | 25 (89%) | 3 (11%) | 28 | 176 (99%) | 1 <1%) | 177 | 60 (92%) | | 5 (8%) | 65 | 144 (99%) | | 2 (1%) | 146 |
| 2 | 4 (57%) | 3 (43%) | 7 | 81 (94%) | 5 (6%) | 86 | 244 (93%) | | 19 (7%) | 263 | 123 (85%) | | 22 (15%) | 145 |
| 3 | 0 | 0 | 0 | 28 (100%) | 0 (0%) | 28 | 69 (78%) | | 19 (22%) | 88 | 45 (74%) | | 16 (26%) | 61 |
| 4 | 4 (44%) | 5 (56%) | 9 | 12 (86%) | 2 (14%) | 14 | 27 (66%) | | 14 (34%) | 41 | 24 (86%) | | 4 (14%) | 28 |
| 5 | 1 (17%) | 5 (83%) | 6 | 5 (83%) | 1 (17%) | 6 | 10 (53%) | | 9 (47%) | 19 | 12 (86%) | | 2 (14%) | 14 |
| 6 | 0 | 0 | 0 | 2 (40%) | 3 (60%) | 5 | 15 (56%) | | 12 (44%) | 27 | 2 (67%) | | 1 (33%) | 3 |
| 7 | 0 (0%) | 1 (100%) | 1 | 1 (50%) | 1 (50%) | 2 | 5 (63%) | | 3 (38%) | 8 | 1 (100%) | | 0 (0%) | 1 |
| 8 | 0 | 0 | 0 | 0 | 0 | 0 | 2 (50%) | | 2 (50%) | 4 | 0 | | 0 | 0 |
| 9 | 0 | 0 | 0 | 0 | 0 | 0 | 2 (67%) | | 1 (33%) | 3 | 0 | | 0 | 0 |
| 10 | 0 | 0 | 0 | 0 (0%) | 1 (100%) | 1 | 0 | | 0 | 0 | 0 | | 0 | 0 |
| 11 | 355 (98%) | 6 (2%) | 361 | 406 (100%) | 2 (<1%) | 408 | 282 (96%) | | 11 (4%) | 293 | 445 (99%) | | 4 (<1%) | 449 |

**Table S4: Distribution of each severity score by each country (Lao PDR, Malawi, Mozambique, and Zimbabwe) among febrile participants (aged ≥15 years) enrolled between 2018 and 2021 with complete data by time of follow-up.** MEWS=modified early warning score, qSOFA=quick sequential organ failure assessment, UVA=universal vital assessment score.

| **Variable** | **Overall, N = 2,797** | **Alive at follow-up, N = 2,612** | **Dead at follow-up, N = 185** | **Unadjusted OR (95% CI)** | **p-value** | **Adjusted OR (95% CI)** | **p-value** |
| --- | --- | --- | --- | --- | --- | --- | --- |
| MEWS |  |  |  |  | <0.001 |  | <0.001 |
| 1 | 599 | 583 (97%) | 16 (3%) | — |  | — |  |
| 2 | 562 | 536 (95%) | 26 (5%) | 1.77 (0.95 - 3.40) |  | 1.91 (1.00 - 3.75) |  |
| 3 | 555 | 529 (95%) | 26 (5%) | 1.79 (0.96 - 3.44) |  | 2.06 (1.08 - 4.03) |  |
| 4 | 405 | 372 (92%) | 33 (8%) | 3.23 (1.78 - 6.10) |  | 3.52 (1.90 - 6.79) |  |
| 5 | 287 | 257 (90%) | 30 (10%) | 4.25 (2.31 - 8.12) |  | 4.26 (2.25 - 8.34) |  |
| 6 | 218 | 201 (92%) | 17 (8%) | 3.08 (1.52 - 6.27) |  | 3.37 (1.62 - 7.06) |  |
| 7 | 116 | 94 (81%) | 22 (19%) | 8.53 (4.35 - 17.1) |  | 9.84 (4.78 - 20.7) |  |
| 8 | 39 | 33 (85%) | 6 (15%) | 6.62 (2.26 - 17.3) |  | 4.99 (1.64 - 13.7) |  |
| 9 | 16 | 7 (44%) | 9 (56%) | 46.8 (15.6 - 147.0) |  | 47.2 (13.8 - 169) |  |
| Age group |  |  |  |  | <0.001 |  | <0.001 |
| 15 - <25 | 784 | 769 (98%) | 15 (2%) | — |  | — |  |
| 25 - <35 | 782 | 740 (95%) | 42 (5%) | 2.91 (1.64 - 5.46) |  | 2.10 (1.14 - 4.05) |  |
| 35 - <40 | 590 | 545 (92%) | 45 (8%) | 4.23 (2.39 - 7.92) |  | 2.32 (1.25 - 4.50) |  |
| 45 - <55 | 301 | 269 (89%) | 32 (11%) | 6.10 (3.31 - 11.7) |  | 4.27 (2.22 - 8.53) |  |
| 55 - <65 | 197 | 169 (86%) | 28 (14%) | 8.49 (4.50 - 16.6) |  | 7.51 (3.79 - 15.4) |  |
| 65+ | 143 | 120 (84%) | 23 (16%) | 9.83 (5.03 - 19.7) |  | 10.9 (5.38 - 22.7) |  |
| Sex |  |  |  |  | <0.001 |  | <0.001 |
| Female | 1,684 | 1,600 (95%) | 84 (5%) | — |  | — |  |
| Male | 1,113 | 1,012 (91%) | 101 (9%) | 1.90 (1.41 - 2.57) |  | 1.93 (1.40 - 2.68) |  |
| HIV status |  |  |  |  | <0.001 |  | <0.001 |
| Negative | 2,112 | 2,039 (97%) | 73 (3%) | — |  | — |  |
| Positive | 685 | 573 (84%) | 112 (16%) | 5.46 (4.02 - 7.46) |  | 4.92 (3.49 - 6.98) |  |

**Table S5: Unadjusted and adjusted logistic regression** results for modified early warning score **(MEWS) among febrile participants (aged ≥15 years) enrolled between 2018 and 2021 with complete data by time of follow-up across four sites (Lao PDR, Malawi, Mozambique, and Zimbabwe)**. OR = Odds ratio, CI = Confidence intervals. Adjusted for age, sex and HIV status, reference is a MEWS score of 1 (rather than 0).

| **Variable** | **Overall, N = 2,797** | **Alive, N = 2,612** | **Dead, N = 185^1^** | **Unadjusted OR** | **p-value** | **Adjusted OR (95% CI)** | **p-value** |
| --- | --- | --- | --- | --- | --- | --- | --- |
| qSOFA score |  |  |  |  | <0.001 |  | <0.001 |
| 0 | 1,436 | 1,387 (97%) | 49 (3%) | — |  | — |  |
| 1 | 1,126 | 1,047 (93%) | 79 (7%) | 2.14 (1.49 - 3.10) |  | 2.13 (1.46 - 3.13) |  |
| 2 | 226 | 176 (78%) | 50 (22%) | 8.04 (5.26 - 12.3) |  | 8.26 (5.24 - 13.1) |  |
| 3 | 9 | 2 (22%) | 7 (78%) | 99.1 (23.3 - 677) |  | 89.1 (18.5 - 658) |  |
| Age group |  |  |  |  | <0.001 |  | <0.001 |
| 15 - <25 | 784 | 769 (98%) | 15 (2%) | — |  | — |  |
| 25 - <35 | 782 | 740 (95%) | 42 (5%) | 2.91 (1.64 - 5.46) |  | 1.99 (1.08 - 3.84) |  |
| 35 - <40 | 590 | 545 (92%) | 45 (8%) | 4.23 (2.39 - 7.92) |  | 2.11 (1.14 - 4.10) |  |
| 45 - <55 | 301 | 269 (89%) | 32 (11%) | 6.10 (3.31 - 11.7) |  | 4.06 (2.12 - 8.11) |  |
| 55 - <65 | 197 | 169 (86%) | 28 (14%) | 8.49 (4.50 - 16.6) |  | 6.34 (3.21 - 13.0) |  |
| 65+ | 143 | 120 (84%) | 23 (16%) | 9.83 (5.03 - 19.7) |  | 8.31 (4.03 - 17.5) |  |
| Sex |  |  |  |  | <0.001 |  | <0.001 |
| Female | 1,684 | 1,600 (95%) | 84 (5%) | — |  | — |  |
| Male | 1,113 | 1,012 (91%) | 101 (9%) | 1.90 (1.41 - 2.57) |  | 2.04 (1.47 - 2.83) |  |
| HIV status |  |  |  |  | <0.001 |  | <0.001 |
| Negative | 2,112 | 2,039 (97%) | 73 (4%) | — |  | — |  |
| Positive | 685 | 573 (84%) | 112 (16%) | 5.46 (4.02 - 7.46) |  | 5.62 (3.98 - 7.99) |  |

**Table S6: Unadjusted and adjusted logistic regression results for quick sequential organ failure assessment (qSOFA) among febrile participants (aged ≥15 years) enrolled between 2018 and 2021 with complete data by time of follow-up across four sites (Lao PDR, Malawi, Mozambique, and Zimbabwe**). OR = Odds ratio, CI = Confidence intervals. Adjusted for age, sex and HIV status.

| **Variable** | **Overall, N = 2,797** | **Alive at follow-up,**  **N = 2,612** | **Dead at follow-up, N = 185** | **Unadjusted OR** | **p-value** | **Adjusted OR (95% CI)** | **p-value** |
| --- | --- | --- | --- | --- | --- | --- | --- |
| UVA score |  |  |  |  | <0.001 |  | <0.001 |
| 0 | 1,511 | 1,488 (98%) | 23 (2%) | — |  | — |  |
| 1 | 416 | 405 (97%) | 11 (3%) | 1.76 (0.82 - 3.55) |  | 2.13 (0.98 - 4.34) |  |
| 2 | 501 | 452 (90%) | 49 (10%) | 7.01 (4.28 - 11.8) |  | 6.55 (3.95 - 11.2) |  |
| 3 | 177 | 142 (80%) | 35 (20%) | 15.9 (9.23 - 28.1) |  | 17.5 (9.92 - 31.5) |  |
| 4 | 92 | 67 (73%) | 25 (27%) | 24.1 (13.0 - 45.0) |  | 24.9 (13.1 - 47.8) |  |
| 5 | 45 | 28 (62%) | 17 (38%) | 39.3 (18.8 - 81.7) |  | 37.0 (17.1 - 80.0) |  |
| 6 | 35 | 19 (54%) | 16 (46%) | 54.5 (24.9 - 120) |  | 56.6 (24.9 - 130) |  |
| 7 | 12 | 7 (58%) | 5 (42%) | 46.2 (12.9 - 156) |  | 47.8 (12.7 - 170) |  |
| 8 - 11 | 8 | 4 (50%) | 4 (50%) | 64.7 (14.5 - 289) |  | 72.4 (15.1 - 346) |  |
| Age group |  |  |  |  | <0.001 |  | <0.001 |
| 15 - <25 | 784 | 769 (98%) | 15 (2%) | — |  | — |  |
| 25 - <35 | 782 | 740 (95%) | 42 (5%) | 2.91 (1.64 - 5.46) |  | 1.95 (1.05 - 3.77) |  |
| 35 - <40 | 590 | 545 (92%) | 45 (8%) | 4.23 (2.39 - 7.92) |  | 1.85 (1.00 - 3.59) |  |
| 45 - <55 | 301 | 269 (89%) | 32 (11%) | 6.10 (3.31 - 11.7) |  | 3.94 (2.03 - 7.94) |  |
| 55 - <65 | 197 | 169 (86%) | 28 (14%) | 8.49 (4.50 - 16.6) |  | 6.12 (3.05 - 12.7) |  |
| 65+ | 143 | 120 (84%) | 23 (16%) | 9.83 (5.03 - 19.7) |  | 8.25 (3.95 - 17.7) |  |
| Sex |  |  |  |  | <0.001 |  | <0.001 |
| Female | 1,684 | 1,600 (95%) | 84 (5%) | — |  | — |  |
| Male | 1,113 | 1,012 (91%) | 101 (9%) | 1.90 (1.41 - 2.57) |  | 1.80 (1.29 - 2.52) |  |

**Table S7: Unadjusted and adjusted logistic regression results for niversal vital assessment score (UVA) among febrile participants (aged ≥15 years) enrolled between 2018 and 2021 with complete data by time of follow-up across four sites (Lao PDR, Malawi, Mozambique, and Zimbabwe).** OR = Odds ratio, CI = Confidence intervals. Adjusted for age, sex and HIV status.

| **Section/Topic** | **Item** | **Checklist Item** | **Page** |
| --- | --- | --- | --- |
| **Title and abstract** | | | |
| Title | 1 | Identify the study as developing and/or validating a multivariable prediction model, the target population, and the outcome to be predicted. | 1 |
| Abstract | 2 | Provide a summary of objectives, study design, setting, participants, sample size, predictors, outcome, statistical analysis, results, and conclusions. | 4 |
| **Introduction** | | | |
| Background and objectives | 3a | Explain the medical context (including whether diagnostic or prognostic) and rationale for developing or validating the multivariable prediction model, including references to existing models. | 5 |
|  | 3b | Specify the objectives, including whether the study describes the development or validation of the model or both. | 5 |
| **Methods** | | | |
| Source of data | 4a | Describe the study design or source of data (e.g., randomized trial, cohort, or registry data), separately for the development and validation data sets, if applicable. | 6 |
|  | 4b | Specify the key study dates, including start of accrual; end of accrual; and, if applicable, end of follow-up. | 6 |
| Participants | 5a | Specify key elements of the study setting (e.g., primary care, secondary care, general population) including number and location of centres. | 6 |
|  | 5b | Describe eligibility criteria for participants. | 6 |
|  | 5c | Give details of treatments received, if relevant. | n/a |
| Outcome | 6a | Clearly define the outcome that is predicted by the prediction model, including how and when assessed. | 7 |
|  | 6b | Report any actions to blind assessment of the outcome to be predicted. | n/a |
| Predictors | 7a | Clearly define all predictors used in developing or validating the multivariable prediction model, including how and when they were measured. | 7 |
|  | 7b | Report any actions to blind assessment of predictors for the outcome and other predictors. | n/a |
| Sample size | 8 | Explain how the study size was arrived at. | 6 |
| Missing data | 9 | Describe how missing data were handled (e.g., complete-case analysis, single imputation, multiple imputation) with details of any imputation method. | 8 |
| Statistical analysis methods | 10a | Describe how predictors were handled in the analyses. | 7 |
|  | 10b | Specify type of model, all model-building procedures (including any predictor selection), and method for internal validation. | 7 |
|  | 10d | Specify all measures used to assess model performance and, if relevant, to compare multiple models. | 7 |
| Risk groups | 11 | Provide details on how risk groups were created, if done. | 7 |
| **Results** | | | |
| Participants | 13a | Describe the flow of participants through the study, including the number of participants with and without the outcome and, if applicable, a summary of the follow-up time. A diagram may be helpful. | 9 |
|  | 13b | Describe the characteristics of the participants (basic demographics, clinical features, available predictors), including the number of participants with missing data for predictors and outcome. | 9 |
| Model development | 14a | Specify the number of participants and outcome events in each analysis. | 10 |
|  | 14b | If done, report the unadjusted association between each candidate predictor and outcome. | 10 |
| Model specification | 15a | Present the full prediction model to allow predictions for individuals (i.e., all regression coefficients, and model intercept or baseline survival at a given time point). | 10 |
|  | 15b | Explain how to the use the prediction model. | n/a |
| Model performance | 16 | Report performance measures (with CIs) for the prediction model. | 11 |
| **Discussion** | | | |
| Limitations | 18 | Discuss any limitations of the study (such as nonrepresentative sample, few events per predictor, missing data). | 13 |
| Interpretation | 19b | Give an overall interpretation of the results, considering objectives, limitations, and results from similar studies, and other relevant evidence. | 14 |
| Implications | 20 | Discuss the potential clinical use of the model and implications for future research. | 15 |
| **Other information** | | | |
| Supplementary information | 21 | Provide information about the availability of supplementary resources, such as study protocol, Web calculator, and data sets. | 8 |
| Funding | 22 | Give the source of funding and the role of the funders for the present study. | 8 |

Table S8: Transparent Reporting of a multivariable prediction model for Individual Prognosis Or Diagnosis (TRIPOD) Checklist: Prediction Model Development.


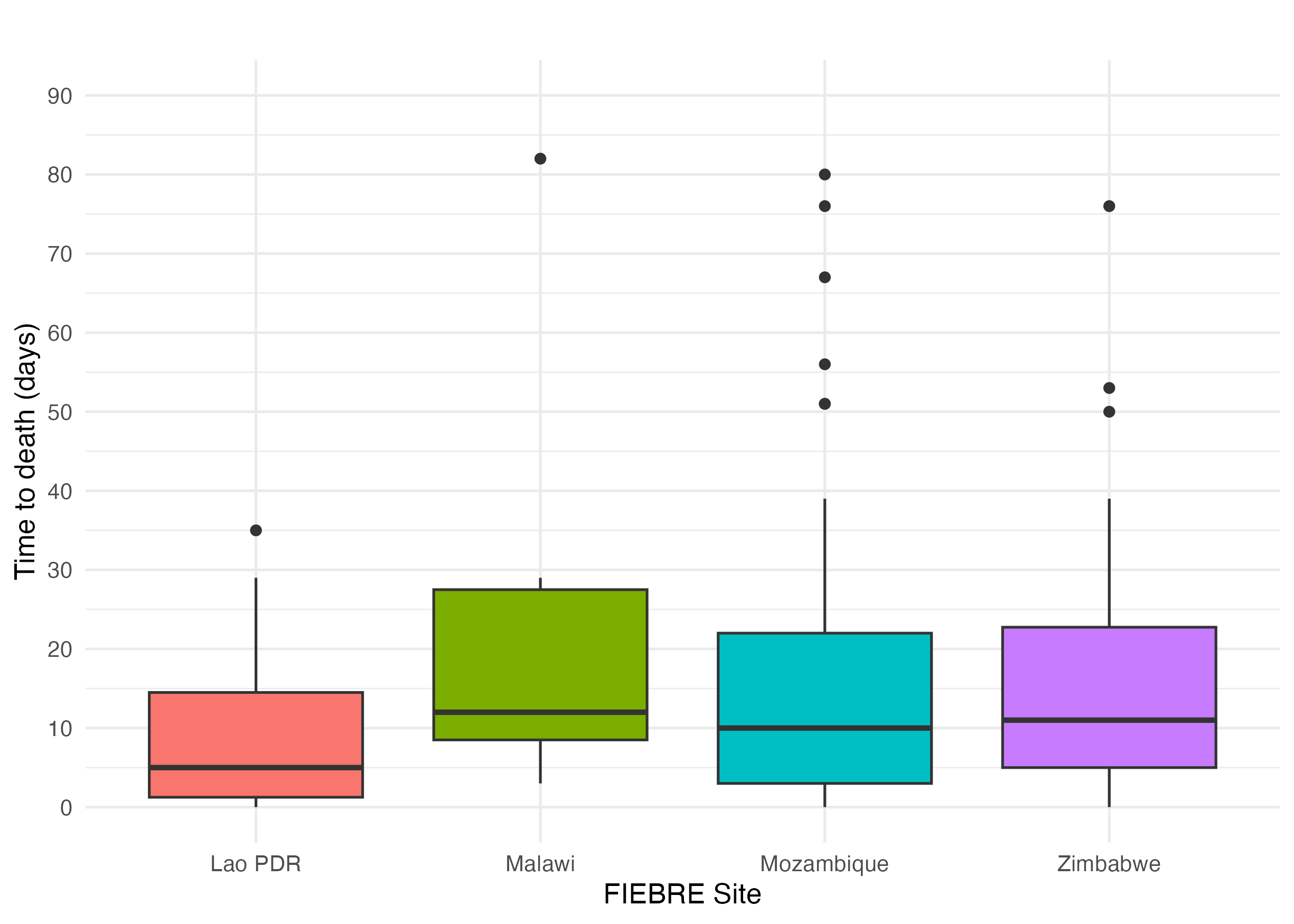


**Figure** **S1: Box and whisker plot of time to death (days) by time to follow-up, by site among febrile participants (aged ≥15 years) enrolled between 2018 and 2021.**


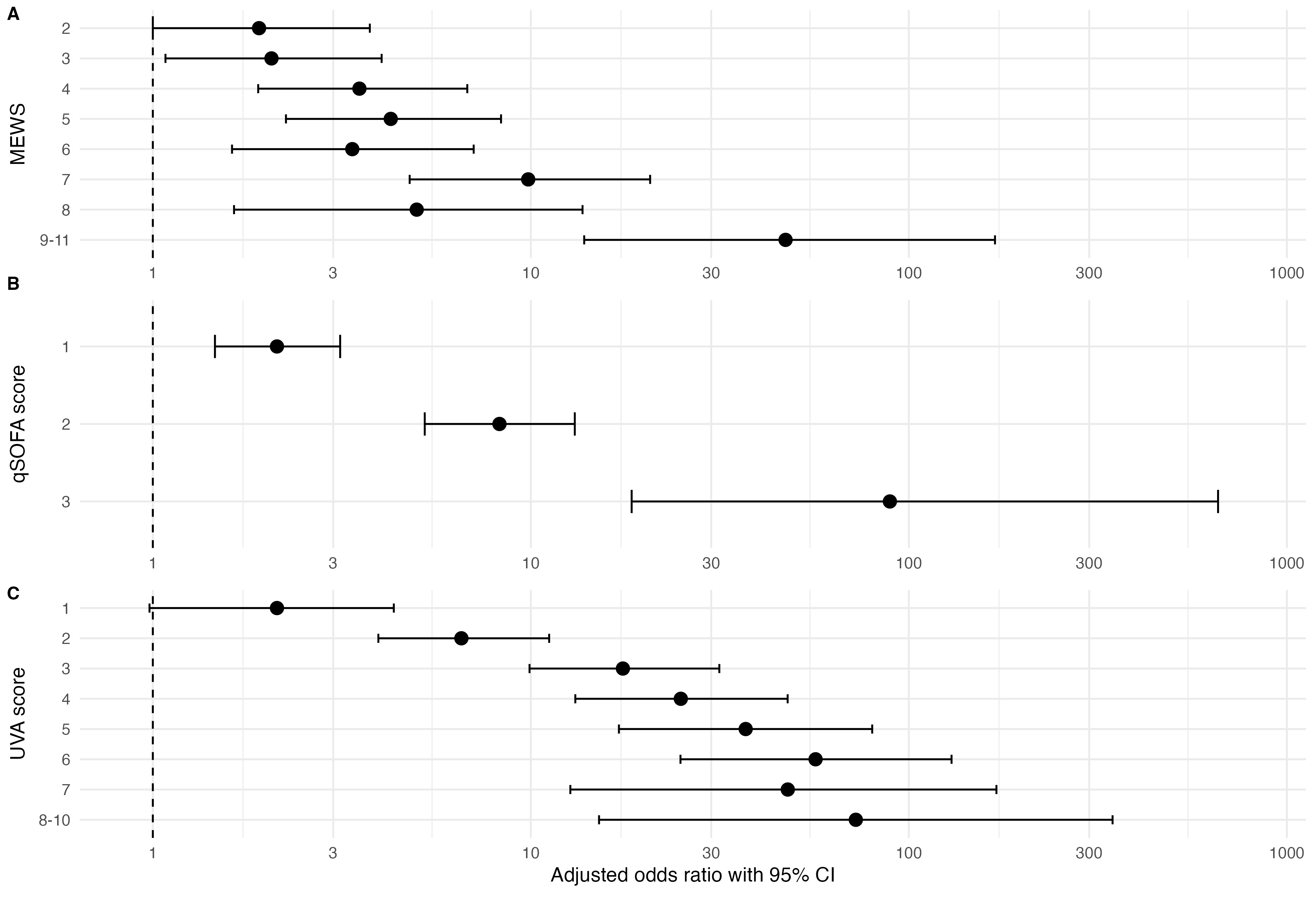


**Figure S2: Adjusted odds ratios for a) MEWS, b) qSOFA and c) UVA severity scores predicting mortality at time of follow-up among febrile participants (aged ≥15 years) enrolled between 2018 and 2021 across four sites (Lao PDR, Malawi, Mozambique, and Zimbabwe**). MEWS=modified early warning score, qSOFA=quick sequential organ failure assessment, UVA=universal vital assessment score, CI= confidence interval.


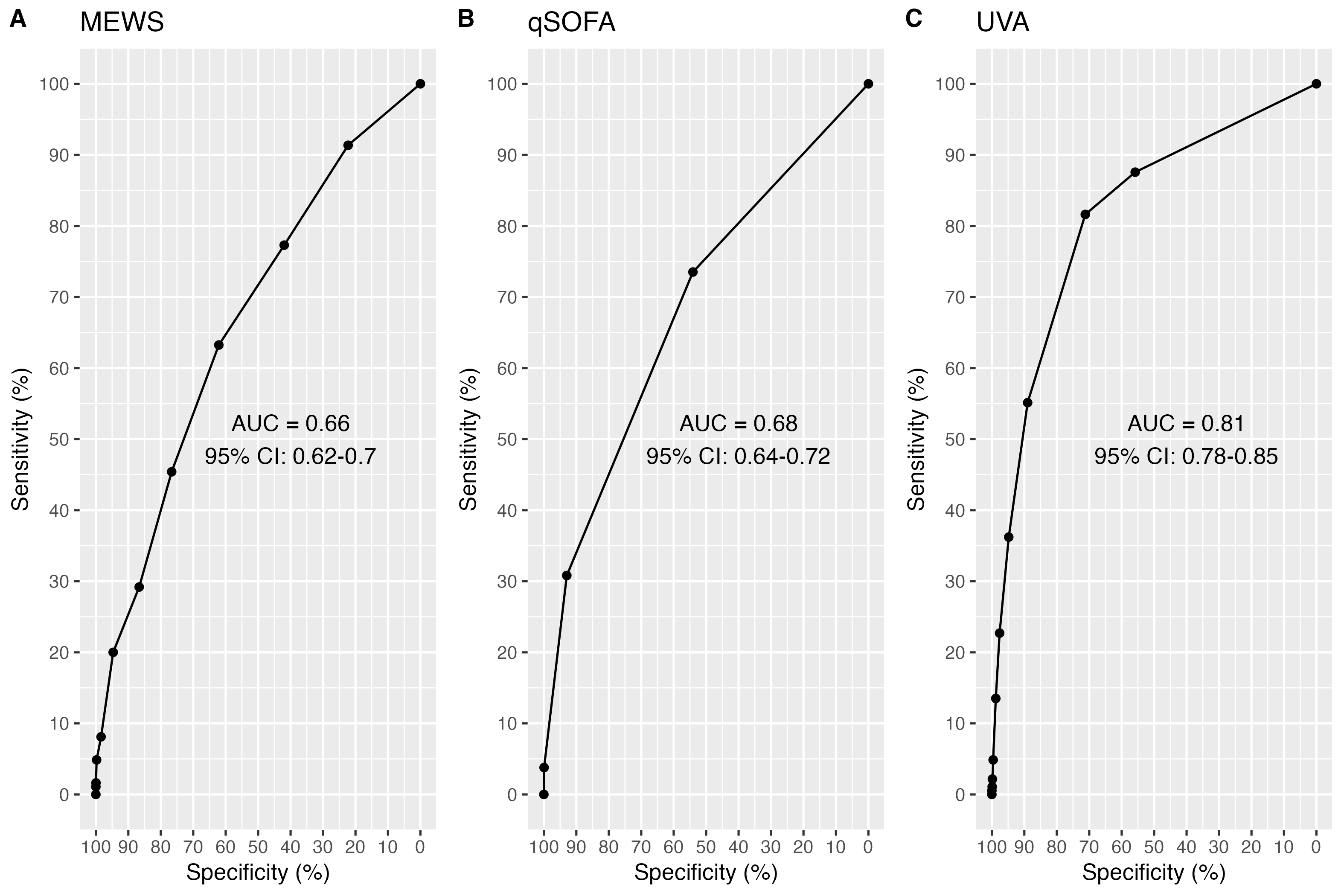


**Figure S3: Sensitivity analysis ROC curves assuming all cases with missing data survived by follow-up, for a) MEWS, b) qSOFA and c) UVA severity scores for predicting mortality by time of follow-up among febrile participants (aged ≥15 years) enrolled between 2018 and 2021 across four sites (Lao PDR, Malawi, Mozambique, and Zimbabwe).** ROC=receiver operating characteristic, AUC=area under the curve, MEWS=modified early warning score, qSOFA=quick sequential organ failure assessment, UVA=universal vital assessment score, CI=confidence interval.


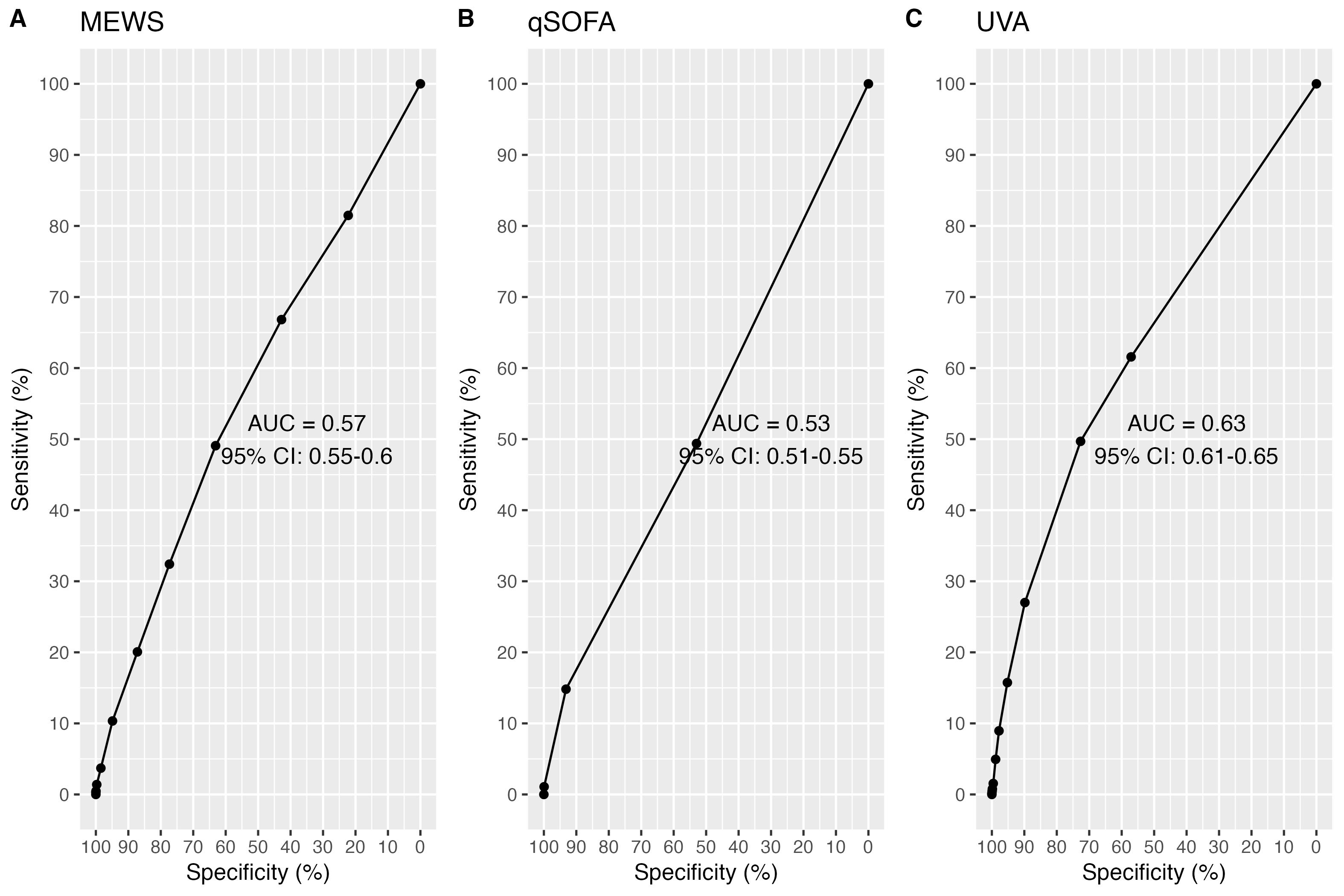


**Figure S4: Sensitivity analysis ROC curves assuming all cases with missing data died by follow-up, for a) MEWS, b) qSOFA and c) UVA severity scores for predicting mortality by time of follow-up among febrile participants (aged ≥15 years) enrolled between 2018 and 2021 across four sites (Lao PDR, Malawi, Mozambique, and Zimbabwe).** ROC=receiver operating characteristic, AUC=area under the curve, MEWS=modified early warning score, qSOFA=quick sequential organ failure assessment, UVA=universal vital assessment score, CI=confidence interval.


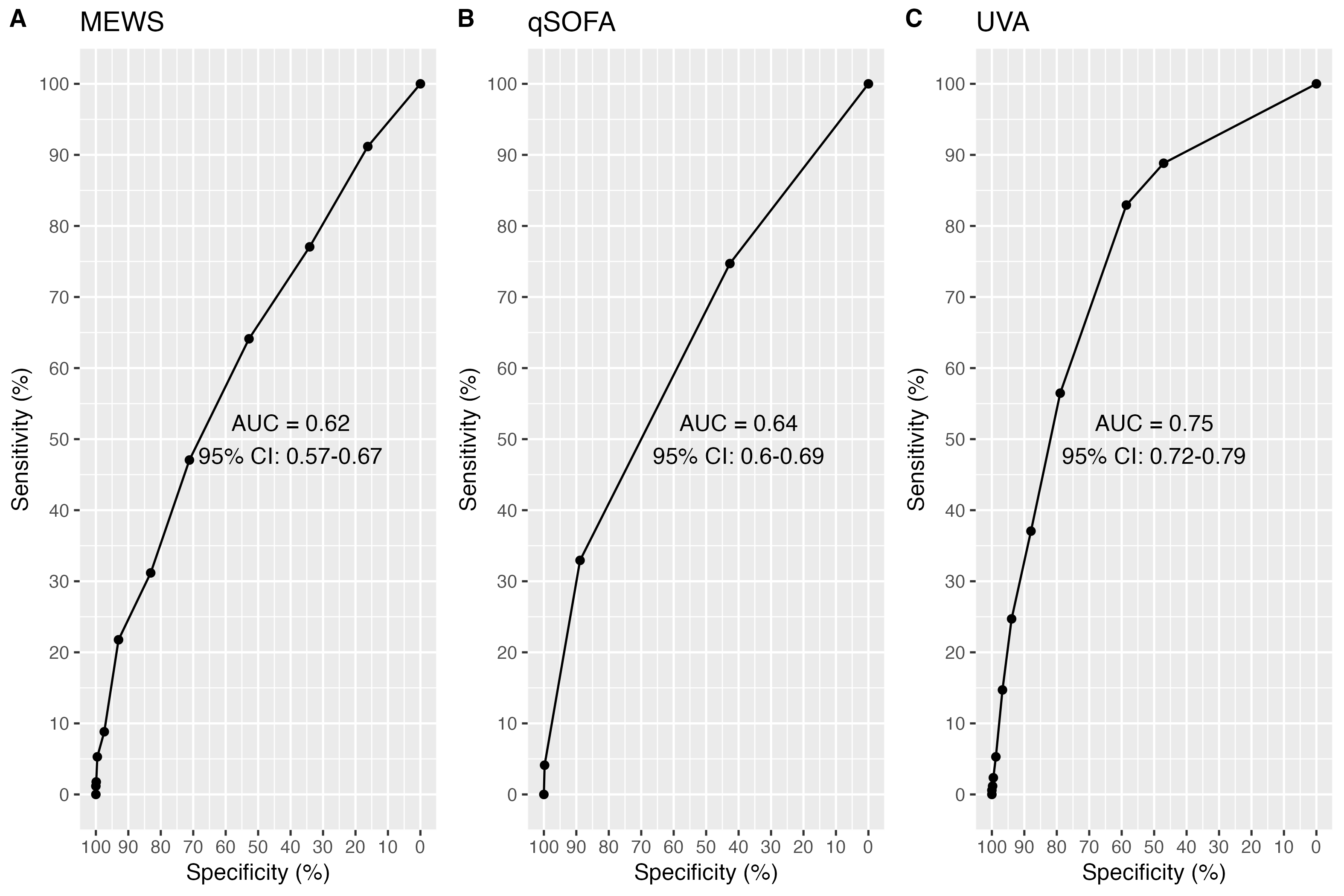


**Figure S5: Inpatient ROC curves for a) MEWS, b) qSOFA and c) UVA severity scores for predicting mortality by time of follow-up among febrile participants (aged ≥15 years) enrolled between 2018 and 2021 across four sites (Lao PDR, Malawi, Mozambique, and Zimbabwe).** ROC=receiver operating characteristic, AUC=area under the curve, MEWS=modified early warning score, qSOFA=quick sequential organ failure assessment, UVA=universal vital assessment score, CI=confidence interval.


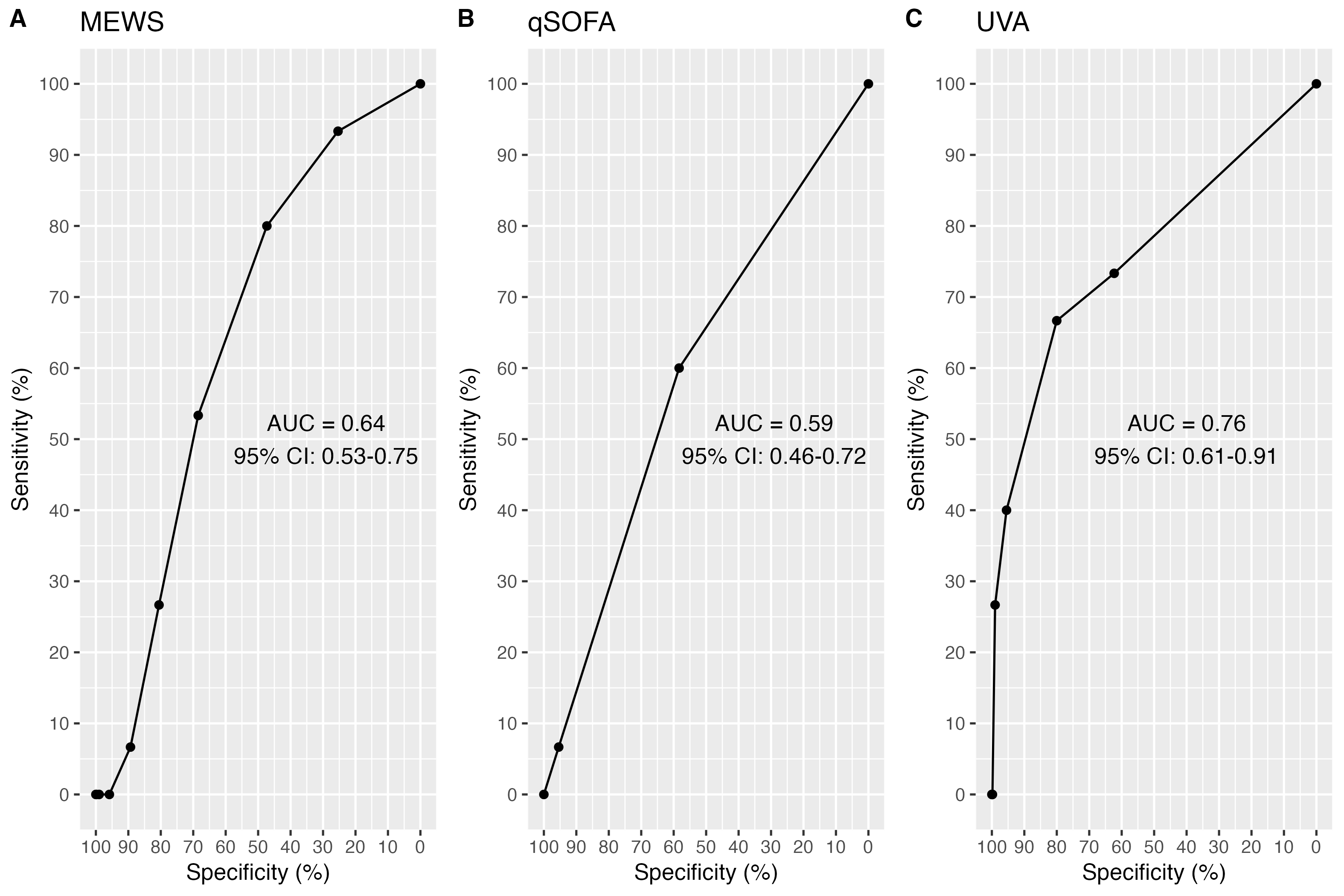


**Figure S6: Outpatient ROC curves for a) MEWS, b) qSOFA and c) UVA severity scores for predicting mortality by time of follow-up among febrile participants (aged ≥15 years) enrolled between 2018 and 2021 across four sites (Lao PDR, Malawi, Mozambique, and Zimbabwe).** ROC=receiver operating characteristic, AUC=area under the curve, MEWS=modified early warning score, qSOFA=quick sequential organ failure assessment, UVA=universal vital assessment score, CI=confidence interval.


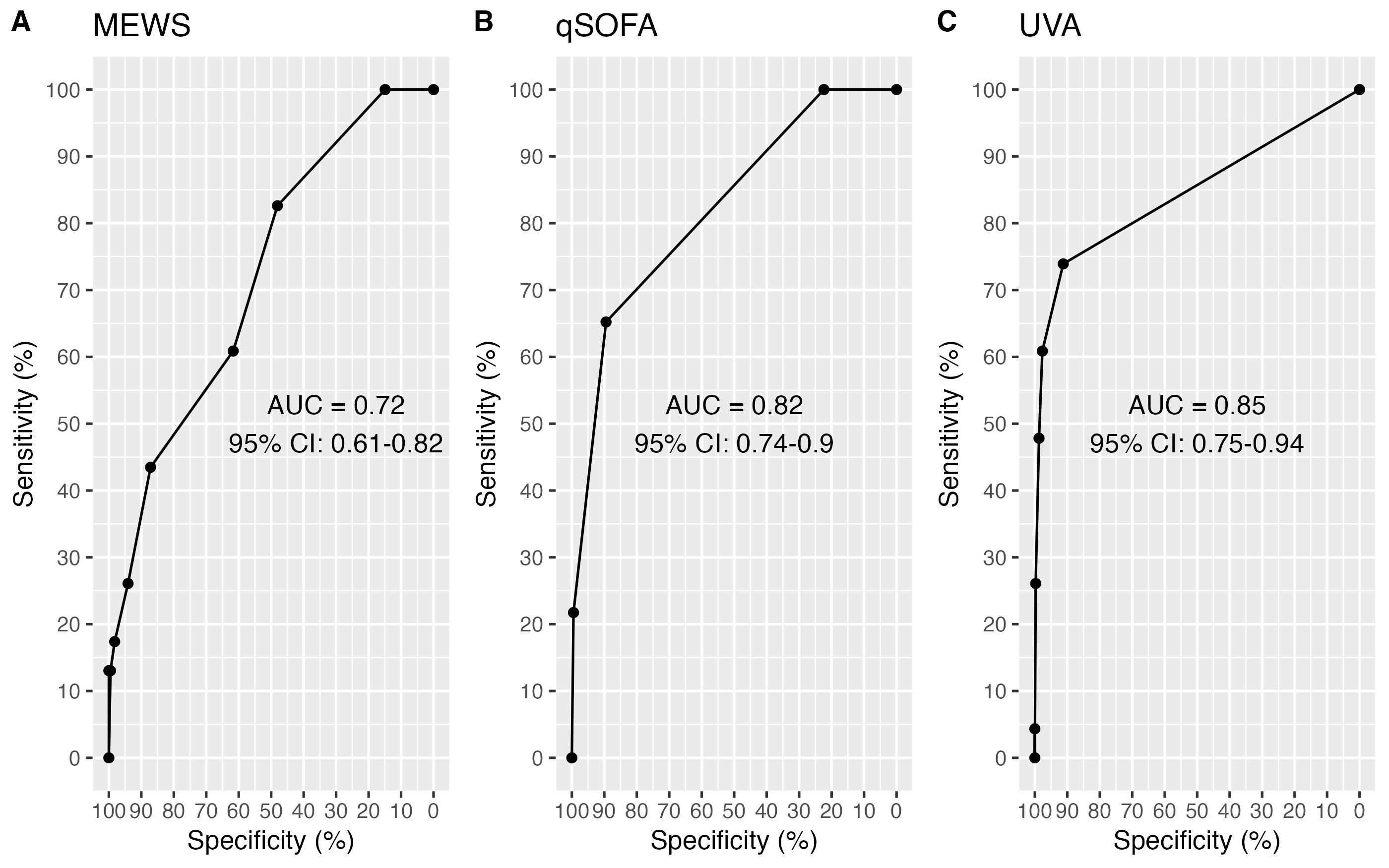


**Figure S7: Lao PDR ROC curves for a) MEWS, b) qSOFA and c) UVA severity scores for predicting mortality by time of follow-up among febrile participants (aged ≥15 years) enrolled between 2018 and 2021.** ROC=receiver operating characteristic, AUC=area under the curve, MEWS=modified early warning score, qSOFA=quick sequential organ failure assessment, UVA=universal vital assessment score, CI=confidence interval.


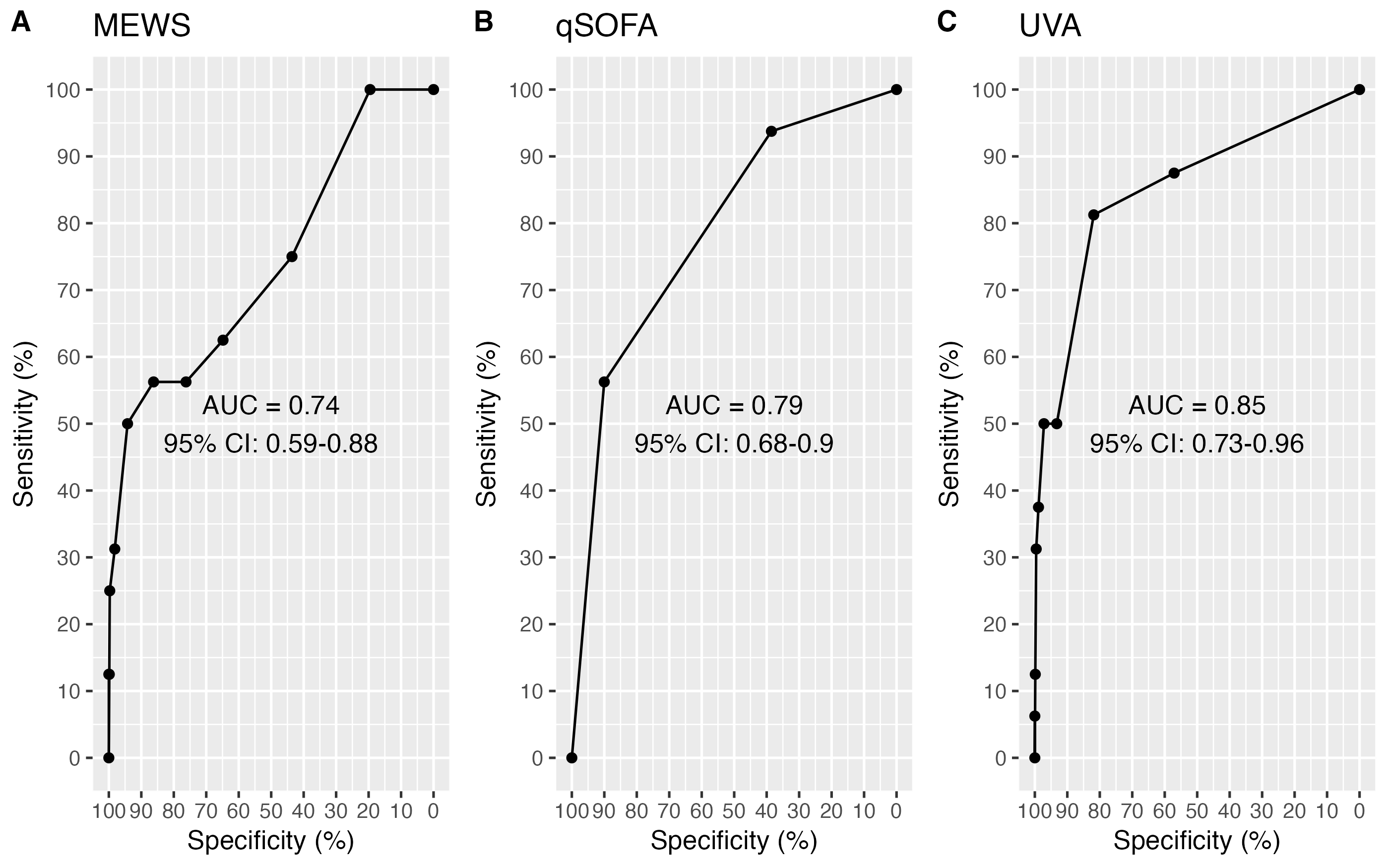


**Figure S8: Malawi ROC curves for a) MEWS, b) qSOFA and c) UVA severity scores for predicting mortality by time of follow-up among febrile participants (aged ≥15 years) enrolled between 2018 and 2021.** ROC=receiver operating characteristic, AUC=area under the curve, MEWS=modified early warning score, qSOFA=quick sequential organ failure assessment, UVA=universal vital assessment score, CI=confidence interval.


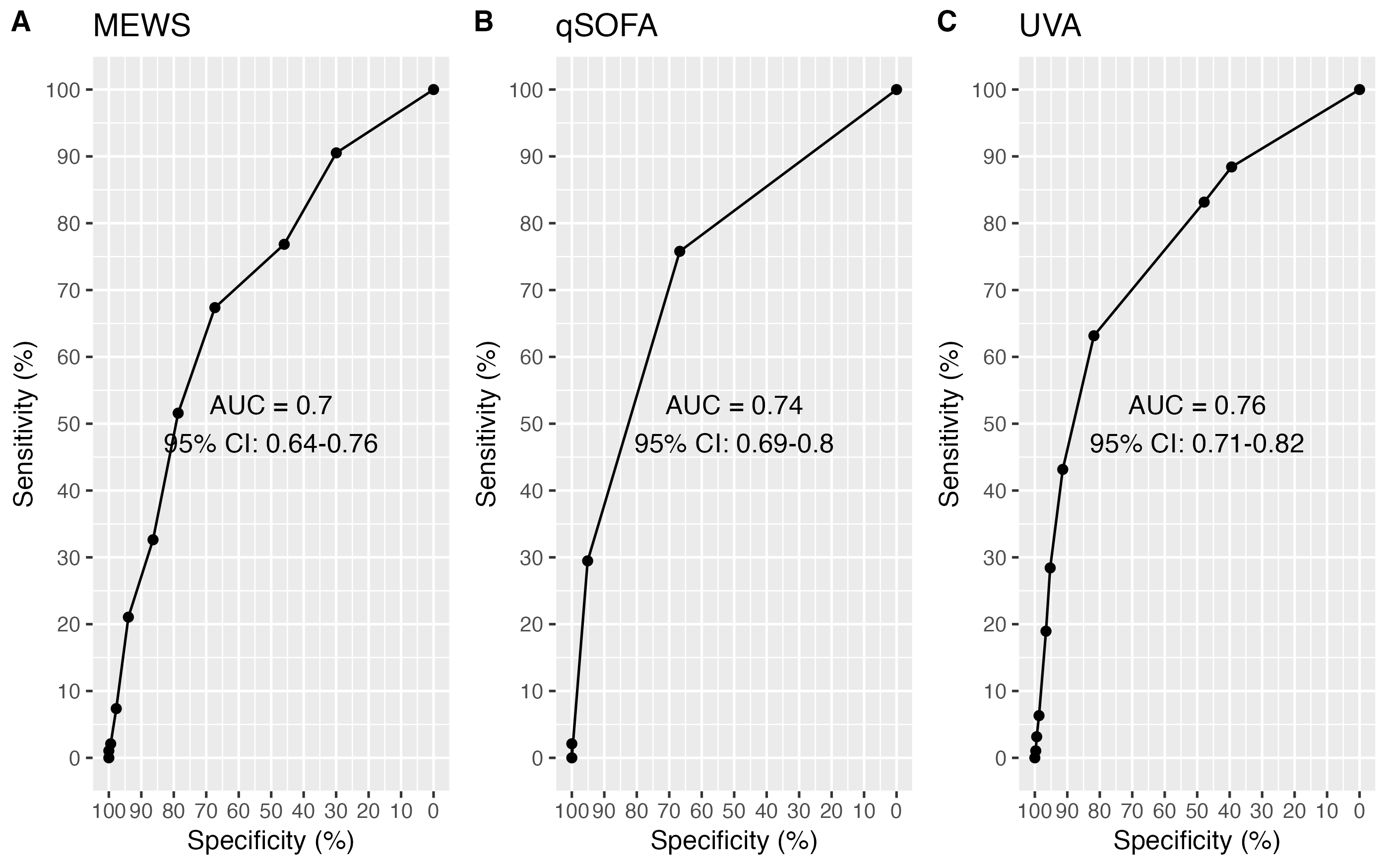


**Figure S9: Mozambique ROC curves for a) MEWS, b) qSOFA and c) UVA severity scores for predicting mortality by time of follow-up among febrile participants (aged ≥15 years) enrolled between 2018 and 2021.** ROC=receiver operating characteristic, AUC=area under the curve, MEWS=modified early warning score, qSOFA=quick sequential organ failure assessment, UVA=universal vital assessment score, CI=confidence interval


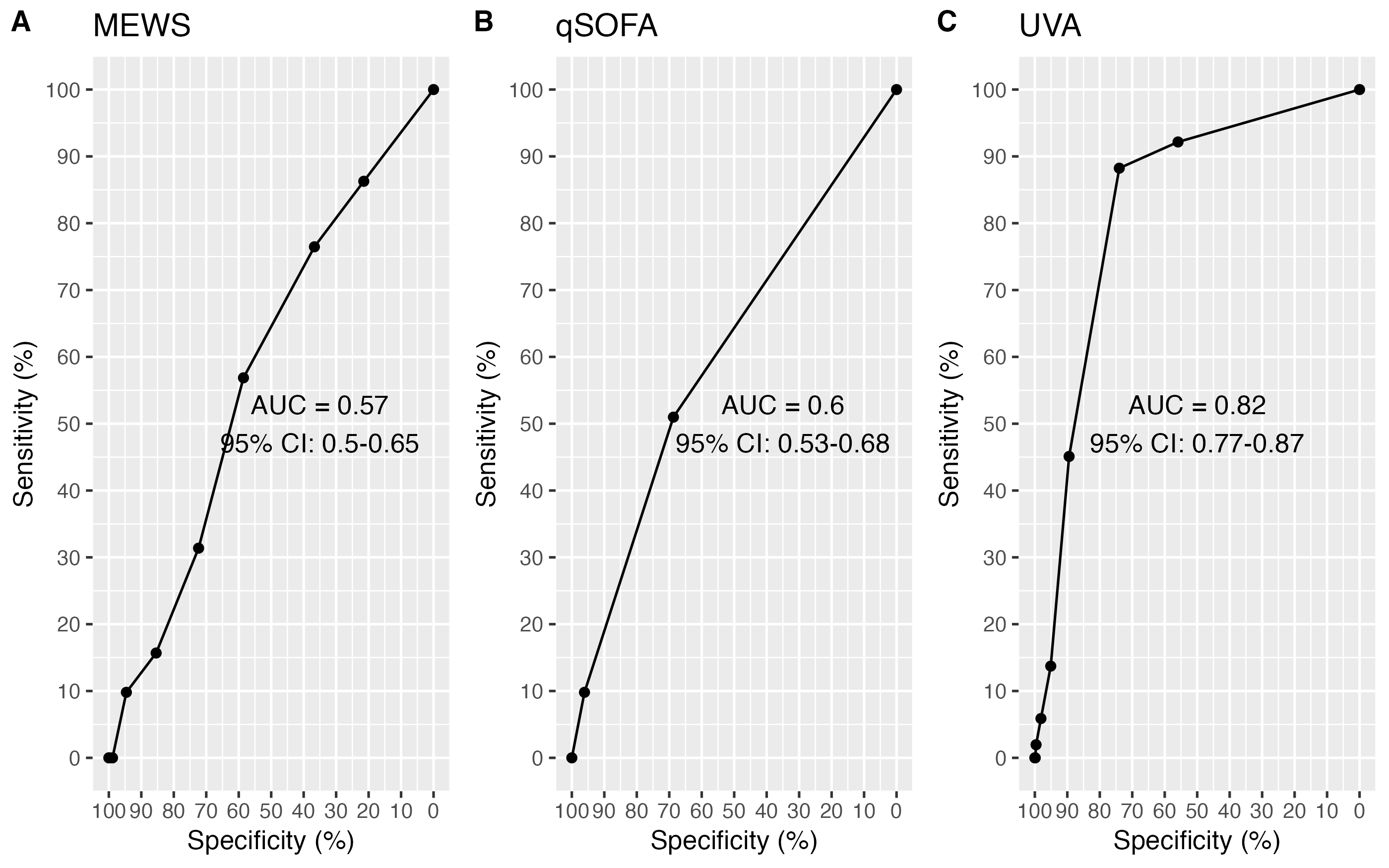


**Figure S10: Zimbabwe ROC curves for a) MEWS, b) qSOFA and c) UVA severity scores for predicting mortality by time of follow-up among febrile participants (aged ≥15 years) enrolled between 2018 and 2021.** ROC=receiver operating characteristic, AUC=area under the curve, MEWS=modified early warning score, qSOFA=quick sequential organ failure assessment, UVA=universal vital assessment score, CI=confidence interval


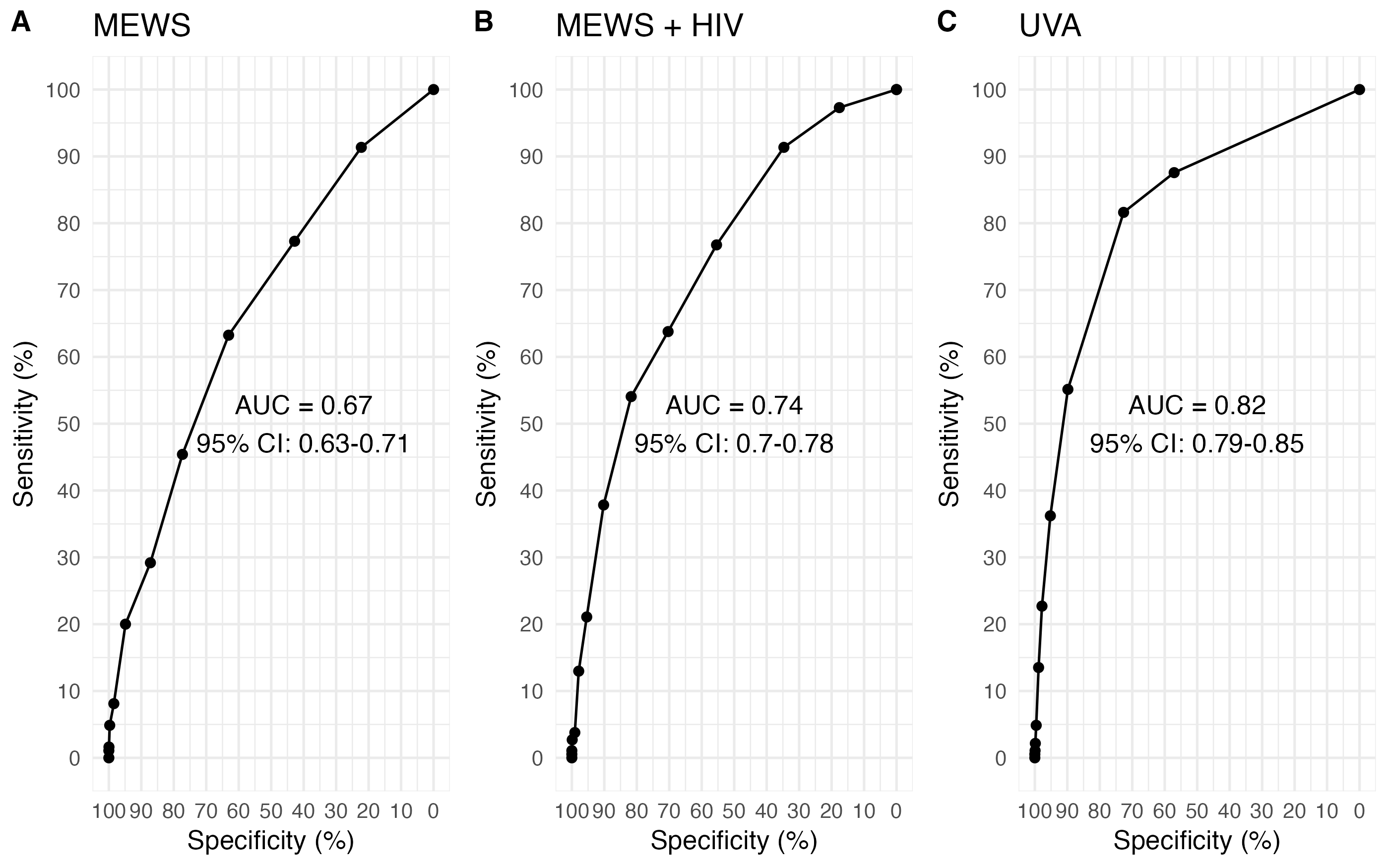


**Figure S11: ROC curves for A) MEWS, B) MEWS + HIV and C) UVA score for predicting mortality by time of follow-up among febrile participants (aged ≥15 years) enrolled between 2018 and 2021 across four sites (Lao PDR, Malawi, Mozambique, and Zimbabwe).** ROC=receiver operating characteristic, AUC=area under the curve, MEWS=modified early warning score, UVA=universal vital assessment score, CI=confidence interval.


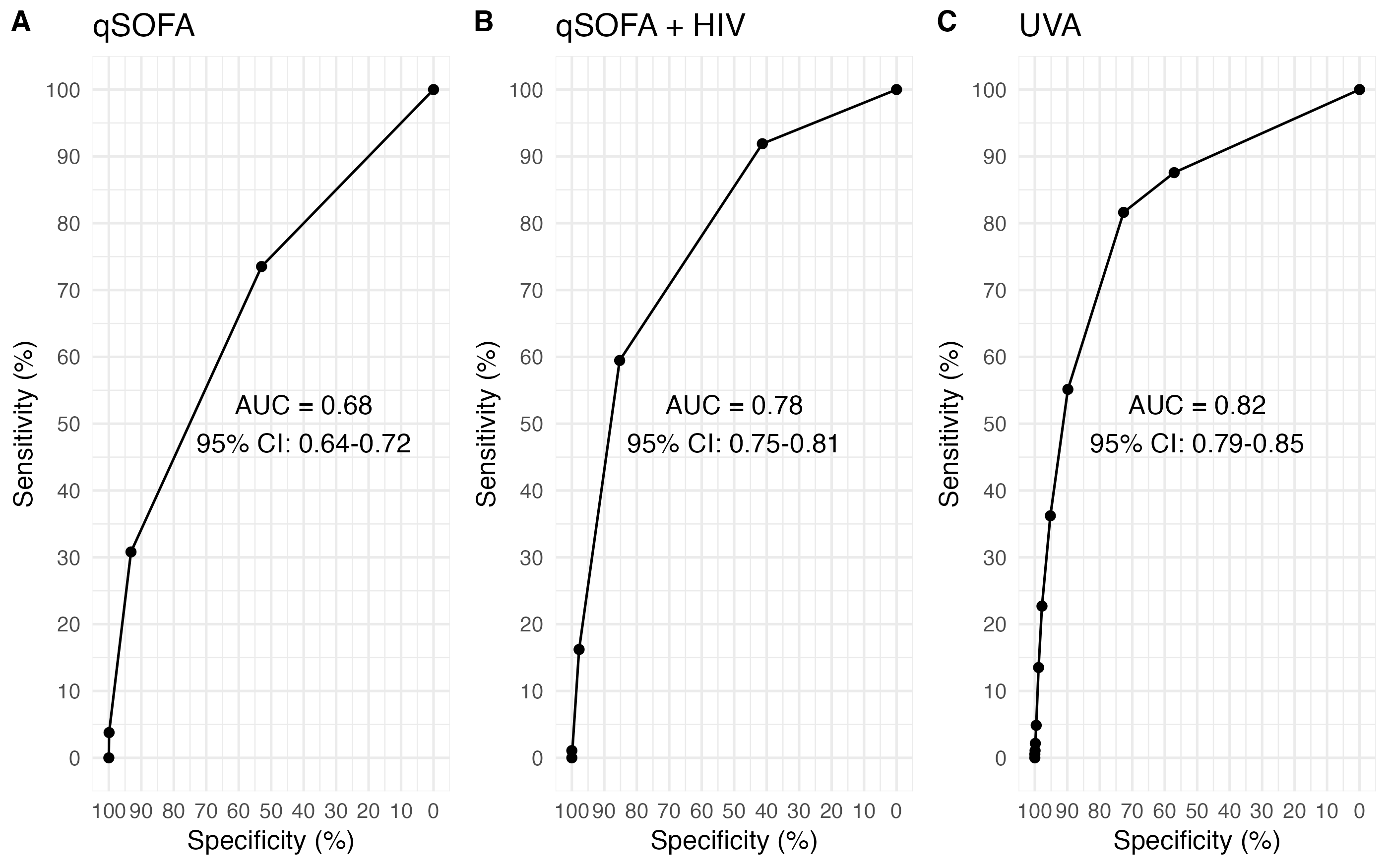


**Figure S12: ROC curves for A) qSOFA, B) qSOFA + HIV and C) UVA score for predicting mortality by time of follow-up among febrile participants (aged ≥15 years) enrolled between 2018 and 2021 across four sites (Lao PDR, Malawi, Mozambique, and Zimbabwe).** ROC=receiver operating characteristic, AUC=area under the curve, qSOFA= quick sequential organ failure assessment, UVA=universal vital assessment score, CI=confidence interval.
