# Supplementary material for: Predicting mortality in febrile adults: comparative performance of the MEWS, qSOFA, and UVA scores using prospectively collected data among patients in four health-care sites in sub-Saharan Africa and South-Eastern Asia
Source: eClinicalMedicine. 2024 Oct 4;77:102856. doi: 10.1016/j.eclinm.2024.102856 (PMC11474423; doi:10.1016/j.eclinm.2024.102856)
Supplement: FIEBRE-consortium-list-for-publications [file mmc2.docx]

FIEBRE Study Consortium

Sara Ajanovic, MSc; ISGlobal, Hospital Clínic - Universitat de Barcelona, Barcelona, Spain

Benjamin Amos, PhD; Independent consultant, Falmouth, Cornwall, UK

Elizabeth A Ashley, MB BS, PhD; Lao-Oxford-Mahosot Hospital-Wellcome Trust Research Unit, Mahosot Hospital, Vientiane, Lao PDR; Centre for Tropical Medicine and Global Health, University of Oxford, Oxford, UK

Oliver Baerenbold, PhD; Faculty of Epidemiology and Population Health, London School of Hygiene & Tropical Medicine, London, UK

Stéphanie Baghoumina; Unité des Virus Émergents, Aix Marseille Univ, IRD 190, INSERM 1207, IHU Méditerranée Infection, 13005, Marseille, France

Núria Balanza, MSc; ISGlobal, Hospital Clínic - Universitat de Barcelona, Barcelona, Spain

Tsitsi Bandason, MSc; Biomedical Research and Training Institute, Harare, Zimbabwe

Quique Bassat, MD, PhD; ISGlobal, Hospital Clínic - Universitat de Barcelona, Barcelona, Spain; Centro de Investigação em Saúde de Manhiça, Maputo, Mozambique; ICREA, Pg. Lluís Companys 23, 08010 Barcelona, Spain and Pediatric Infectious Diseases Unit, Pediatrics Department, Hospital Sant Joan de Déu (University of Barcelona), Barcelona, Spain

Tapan Bhattacharyya, PhD; Department of Infection Biology, Faculty of Infectious and Tropical Diseases, London School of Hygiene & Tropical Medicine, London, UK

Stuart D Blacksell, PhD; Lao-Oxford-Mahosot Hospital-Wellcome Trust Research Unit, Mahosot Hospital, Vientiane, Lao PDR

Zumilda Boca; Centro de Investigação em Saúde de Manhiça, Maputo, Mozambique

Christian Bottomley, PhD; Department of Infectious Disease Epidemiology and International Health, Faculty of Epidemiology and Population Health, London School of Hygiene & Tropical Medicine, London, UK

John Bradley, PhD; MRC International Statistics and Epidemiology Group, Faculty of Epidemiology and Population Health, London School of Hygiene & Tropical Medicine, London, UK

Justina M Bramugy; Centro de Investigação em Saúde de Manhiça, Maputo, Mozambique

Clare IR Chandler, PhD; Department of Global Health and Development, London School of Hygiene & Tropical Medicine, London, UK

Vilada Chansamouth; Lao-Oxford-Mahosot Hospital-Wellcome Trust Research Unit, Mahosot Hospital, Vientiane, Lao PDR

Mabvuto Chimenya; Malawi-Liverpool-Wellcome Trust Clinical Research Programme, Blantyre, Malawi

Joseph Chipanga, BSc; Biomedical Research and Training Institute, Harare, Zimbabwe

Anelsio Cossa; Centro de Investigação em Saúde de Manhiça, Maputo, Mozambique

John A Crump, MB ChB, MD, DTM&H; Centre for International Health, University of Otago, Dunedin, New Zealand

Ethel Dauya; Biomedical Research and Training Institute, Harare, Zimbabwe

Catherine Davis; Micropathology Ltd, University of Warwick Science Park, Coventry, UK

Xavier de Lamballerie; Unité des Virus Émergents, Aix Marseille Univ, IRD 190, INSERM 1207, IHU Méditerranée Infection, 13005, Marseille, France

Justin Dixon, PhD; Department of Global Health and Development, London School of Hygiene & Tropical Medicine, London, UK

Somyoth Douangphachanh; Vientiane Provincial Hospital

Audrey Dubot-Pérès, PhD; Unité des Virus Émergents, Aix Marseille Univ, IRD 190, INSERM 1207, IHU Méditerranée Infection, 13005, Marseille, France

Michelle M Durkin; MiraVista Diagnostics, Indianapolis, Indiana, USA

Nicholas A Feasey, PhD; Department of Clinical Sciences, Liverpool School of Tropical Medicine, Liverpool, UK; Malawi-Liverpool-Wellcome Trust Clinical Research Programme, Blantyre, Malawi; Faculty of Infectious and Tropical Diseases, London School of Hygiene & Tropical Medicine, London, UK

Rashida A Ferrand, MB BS, PhD; Faculty of Infectious and Tropical Diseases, London School of Hygiene & Tropical Medicine, London, UK

Colin Fink; Micropathology Ltd, University of Warwick Science Park, Coventry, UK

Elizabeth J A Fitchett, MRCPCH MPH; Faculty of Infectious and Tropical Diseases, London School of Hygiene & Tropical Medicine, London, UK; Institut Pasteur Dakar, Dakar, Senegal

Alessandro Gerada, MD MRCP FRCPath; Liverpool Clinical Laboratories, Liverpool, UK

Stephen R Graves, MB BS, PhD; Australian Rickettsial Reference Laboratory, University Hospital Geelong, Geelong, Australia

Edward Green, MBBS, MRCP; Department of Clinical Sciences, Liverpool School of Tropical Medicine, Liverpool, UK; Malawi-Liverpool-Wellcome Trust Clinical Research Programme, Blantyre, Malawi

Becca L Handley; Faculty of Infectious and Tropical Diseases, London School of Hygiene & Tropical Medicine, London, UK

Heidi Hopkins, MD, MPH; Faculty of Infectious and Tropical Diseases, London School of Hygiene & Tropical Medicine, London, UK

Coll D Hutchison; Department of Global Health and Development, London School of Hygiene & Tropical Medicine, London, UK

Risara Jaksuwan; Lao-Oxford-Mahosot Hospital-Wellcome Trust Research Unit, Mahosot Hospital, Vientiane, Lao PDR

Jessica Jervis; Liverpool Clinical Laboratories, Liverpool, UK

Jayne Jones; Clinical Diagnostic Parasitology Laboratory, Liverpool School of Tropical Medicine, Liverpool, UK

Kevin C Kain, MD; Sandra Rotman Centre for Global Health, MaRS Centre, Department of Medicine, University Health Network-Toronto General Hospital, University of Toronto, Ontario, Canada; Division of Infectious Diseases, University Health Network, Toronto, Ontario, Canada; Department of Laboratory Medicine and Pathobiology, University of Toronto, Toronto, Ontario, Canada

Suzanne H. Keddie, MSc; Faculty of Epidemiology and Population Health, London School of Hygiene & Tropical Medicine, London, UK

Khamxeng Khounpaseuth; Lao-Oxford-Mahosot Hospital-Wellcome Trust Research Unit, Mahosot Hospital, Vientiane, Lao PDR

Katharina Kranzer, PhD; Faculty of Infectious and Tropical Diseases, London School of Hygiene & Tropical Medicine, London, UK

Khamfong Kunlaya; Lao-Oxford-Mahosot Hospital-Wellcome Trust Research Unit, Mahosot Hospital, Vientiane, Lao PDR

Pankaj Lal; Liverpool Clinical Laboratories, Liverpool, UK

Sham Lal, PhD; Faculty of Infectious and Tropical Diseases, London School of Hygiene & Tropical Medicine, London, UK

David G Lalloo, FRCP; Department of Clinical Sciences, Liverpool School of Tropical Medicine, Liverpool, UK

Manophab Luangraj, MD; Lao-Oxford-Mahosot Hospital-Wellcome Trust Research Unit, Mahosot Hospital, Vientiane, Lao PDR

Yoel Lubell, PhD; Centre for Tropical Medicine and Global Health, University of Oxford, Oxford, UK

David CW Mabey, DM; Faculty of Infectious and Tropical Diseases, London School of Hygiene & Tropical Medicine, London, UK

Eleanor MacPherson, PhD; University of Glasgow, UK

Forget Makoga; Biomedical Research and Training Institute, Harare, Zimbabwe

Sengchanh Manichan; Vientiane Provincial Hospital

Tegwen Marlais, PhD; Faculty of Infectious and Tropical Diseases, London School of Hygiene & Tropical Medicine, London, UK

Florian Maurer, MD; National and WHO Supranational Reference Center for Mycobacteria, Research Center Borstel, Borstel, Germany

Mayfong Mayxay; Lao-Oxford-Mahosot Hospital-Wellcome Trust Research Unit, Mahosot Hospital, Vientiane, Lao PDR

Michael Miles; Department of Infection Biology, Faculty of Infectious and Tropical Diseases, London School of Hygiene & Tropical Medicine, London, UK

Polycarp Mogeni, PhD; Faculty of Infectious and Tropical Diseases, London School of Hygiene & Tropical Medicine, London, UK

Campos Mucasse; Centro de Investigação em Saúde de Manhiça, Maputo, Mozambique

Paul N Newton, DPhil, FRCP; Centre for Tropical Medicine and Global Health, University of Oxford, Oxford, UK

Chelsea Nguyen; Australian Rickettsial Reference Laboratory, University Hospital Geelong, Geelong, Australia

Ioana D Olaru, MSc, PhD; Faculty of Infectious and Tropical Diseases, London School of Hygiene & Tropical Medicine, London, UK

Vilayouth Phimolsarnnousith; Lao-Oxford-Mahosot Hospital-Wellcome Trust Research Unit, Mahosot Hospital, Vientiane, Lao PDR

Mathieu Picardeau; Biology of Spirochetes unit, Institut Pasteur, Paris, France

Chrissy h Roberts, PhD; Faculty of Infectious and Tropical Diseases, London School of Hygiene & Tropical Medicine, London, UK

Amphone Sengduangphachanh; Microbiology Laboratory, Lao-Oxford-Mahosot Hospital-Wellcome Trust Research Unit, Mahosot Hospital, Vientiane, Lao PDR

Siho Sengsavang; Vientiane Provincial Hospital /provincial health office

Molly Sibanda; Biomedical Research and Training Institute, Harare, Zimbabwe

Somvai Singha; UHS, Lao PDR

John Stenos; Australian Rickettsial Reference Laboratory, University Hospital Geelong, Geelong, Australia

Ampai Tanganuchitcharnchai, MSc; Mahidol-Oxford Tropical Medicine Research Unit, Mahidol University, Bangkok, Thailand

Hira Tanvir; Faculty of Infectious and Tropical Diseases, London School of Hygiene & Tropical Medicine, London, UK

James E Ussher, MBChB, PhD; Southern Community Laboratories, Dunedin, New Zealand

Marta Valente; ISGlobal, Hospital Clínic - Universitat de Barcelona, Barcelona, Spain

Marie A Voice, PhD; Micropathology Ltd, University of Warwick Science Park, Coventry, UK

Manivanh Vongsouvath; Microbiology Laboratory, Lao-Oxford-Mahosot Hospital-Wellcome Trust Research Unit, Mahosot Hospital, Vientiane, Lao PDR

Msopole Wamaka; Ministry of Health, Malawi

L Joseph Wheat, MD; MiraVista Diagnostics, Indianapolis, Indiana, USA

Shunmay Yeung, MBBS, PhD; Faculty of Infectious and Tropical Diseases, London School of Hygiene & Tropical Medicine, London, UK
